# Associations Between Early Life Adversity, Reproduction-Oriented Life Strategy, and Borderline Personality Disorder

**DOI:** 10.1001/jamapsychiatry.2023.0694

**Published:** 2023-04-26

**Authors:** Axel Baptista, Valérian Chambon, Nicolas Hoertel, Mark Olfson, Carlos Blanco, David Cohen, Pierre O. Jacquet

**Affiliations:** 1 Institut Jean Nicod, Département d’Études Cognitives, ENS, EHESS, CNRS, PSL University, Paris, France; 2 Université Paris Cité, Paris, France; 3 Service de Psychiatrie de l’Enfant et de l’Adolescent, GH Pitié-Salpêtrière Charles Foix, Assistance Publique-Hôpitaux de Paris (AP-HP), Paris, France; 4 Centre de Recherche en Épidémiologie et en Santé des Populations, INSERM U1018, UVSQ, Université Paris-Saclay, Paris, France; 5 Département Médico-Universitaire Psychiatrie et Addictologie, Assistance Publique-Hôpitaux de Paris (AP-HP), Hôpital Corentin-Celton, Issy-les-Moulineaux, France; 6 INSERM UMR 894, Psychiatry and Neurosciences Center; Paris University, Paris, France; 7 Department of Psychiatry, Columbia University/New York State Psychiatric Institute, New York; 8 Division of Epidemiology, Services, and Prevention Research, National Institute on Drug Abuse, Bethesda, Maryland; 9 Institut des Systèmes Intelligents et de Robotique, Sorbonne Université, ISIR CNRS UMR, Paris, France; 10 Institut du Psychotraumatisme de l’Enfant et de l’Adolescent, Conseil Départemental Yvelines et Hauts-de-Seine et Centre Hospitalier de Versailles, Versailles, France; 11 Département d’études Cognitives, LNC2, INSERM U960, École Normale Supérieure, PSL Research University, Paris, France

## Abstract

**Importance:**

Borderline personality disorder (BPD) is often accompanied by a history of high-risk sexual behavior and somatic comorbidities. Yet, these features are most often considered in isolation and little is known about their underlying developmental pathways. Life history theory, a leading framework in evolutionary developmental biology, can help make sense of the wide range of behaviors and health issues found in BPD.

**Objective:**

To examine whether the emergence of BPD is associated with the prioritization of immediate reproductive goals over longer-term somatic maintenance goals, a life strategy that can be viewed as a developmental response to adverse early life experiences, providing rapid reproductive benefits despite costs to health and well-being.

**Design, Setting, and Participants:**

This study used cross-sectional data from the second wave of the National Epidemiologic Survey on Alcohol and Related Conditions in 2004-2005 (n = 34 653). Civilian, noninstitutionalized individuals in the US, 18 years or older, and those with and without a *DSM-IV* diagnosis of BPD were included. Analysis took place between August 2020 and June 2021.

**Main Outcomes and Measures:**

Structural equation models were used to examine whether early life adversity was associated with the likelihood of a BPD diagnosis, either directly or indirectly through a life strategy whereby individuals trade somatic maintenance for immediate reproduction.

**Results:**

Analyses were performed on a sample of 30 149 participants (females: 17 042 [52%]; mean [SE] age, 48.5 [0.09]; males: 12 747 [48%]; mean [SE] age, 47 [0.08]). Of these, 892 (2.7%) had a diagnosis of BPD and 29 257 (97.3%) did not have BPD. Mean early life adversity, metabolic disorder score, and body mass index were significantly higher among participants with a diagnosis of BPD. In an analysis adjusted for age, individuals with BPD reported having significantly more children than those without BPD (b =0.06; SE, 0.01; *t* = 4.09; *P* < .001). Having experienced greater levels of adversity in early life was significantly associated with a greater risk of being diagnosed with BPD later in life (direct relative risk = 0.268; SE, 0.067; *P* < .001). Importantly, this risk was further increased by 56.5% among respondents who prioritized short-term reproductive goals over somatic maintenance (indirect relative risk = 0.565; SE, 0.056; *P* < .001). Similar patterns of associations were found in male and female individuals.

**Conclusions and Relevance:**

The hypothesis of a reproduction/maintenance life history trade-off mediating the association between early life adversity and BPD helps make sense of the high dimensionality that characterizes the physiological and behavioral correlates of BPD. Additional studies are needed to confirm these results using longitudinal data.

## Key Points

**Question:**

Is borderline personality disorder (BPD) favored by prioritizing immediate reproductive goals over long-term somatic maintenance goals in response to early life adversity?

**Findings:**

In this cross-sectional study of more than 30 000 adults, the association of early life adversity with the risk of being diagnosed with BPD later in life was significantly mediated by an allocation trade-off favoring immediate reproduction over somatic maintenance.

**Meaning:**

BPD may be the psychobehavioral expression of a broader coping strategy whereby individuals deal with adversity by prioritizing the development of reproductive traits and behaviors.

## Teaser

This cross-sectional study examines whether the emergence of borderline personality disorder is associated with the prioritization of immediate reproductive goals over longer-term somatic maintenance goals.

## Introduction

Borderline personality disorder (BPD) emerges by late adolescence or early adulthood and is highly prevalent in clinical and community samples.^1,2^ It includes a wide range of symptoms, from unstable interpersonal relationships, fear of abandonment, emotion dysregulation, feelings of emptiness, and chronic dysphoria or depression. These symptoms are often accompanied by behaviors that significantly impair psychosocial functioning such as substance use,^3,4^ sexual risk-taking,^5,6,7^ low prosociality,^8,9^ interpersonal violence, as well as self-harm,^5,10,11,12^ including suicide attempts.^13,14^ In addition, individuals with BPD have greater prevalence of somatic comorbidity than individuals with any other personality disorders, which contributes to a shortened life span.^2,15,16^ The co-occurrence of these seemingly unrelated manifestations makes BPD not well understood.

Substantial effort has been made to delineate the developmental origins of BPD and better identify its early environmental determinants to aid its prevention. It is commonly accepted that BPD is partly rooted in early life, via exposure to adverse events that include all forms of instability, deprivation, neglect, abuses, or violence occurring within or outside the family household.^17,18,19^ Despite these important advances, little is known about who is at risk of developing BPD.^17^

To better characterize this risk requires consideration of each individual's ability to respond to early life experiences through developmental changes.^20,21^ There is evidence of an association between the mortality risks present in an environment and variations in how individuals organize their life cycles to optimize achievement of their priority biological goals, ie, growth and maintenance, social goals, and reproductive goals,^22^ given limited energetic resources.^23^ From a biological perspective, it makes sense for organisms to outweigh such risks by allocating more resources to the development of behavioral traits that provide rapid reproductive benefits and fewer resources to somatic maintenance traits that provide longer-term survival benefits.^24^

The existing literature indicates that individuals with BPD may exhibit such trade-off in a way that does not involve conscious decision-making. Thus, they enter sexual life at a younger age than individuals without BPD, have more sexual partners, more unprotected sex, and, for women in particular, become parents at a younger age and experience more unintended pregnancies,^3,5,6,25,26,27^ a set of behaviors that is often accompanied by adverse health effects and general medical comorbidities.^16,28,29^

These observations, viewed through the lens of life history theory, a major framework in evolutionary developmental biology, suggest that BPD may facilitate reproductive goals that provide immediate fitness benefits^30,31^ and that it may develop to counteract risks estimated from early life experiences.^32^ Therefore, we tested 3 complementary hypotheses: (1) somatic maintenance traits and short-term reproductive behaviors are negatively correlated through a latent factor representing the resource allocation trade-off described above; (2) adversity experienced early in life is associated with increased risk of BPD expression in adulthood; and (3) the association of early life adversity with the risk of developing BPD is exacerbated for individuals who trade somatic maintenance for short-term reproductive goals. To this end, we used structural equation models (SEMs) coupled with *k*-fold cross-validation analyses on a large nationally representative sample, the National Epidemiological Survey on Alcohol and Related Conditions (NESARC).

## Methods

This study followed the Strengthening the Reporting of Observational Studies in Epidemiology (STROBE) reporting guideline.^33^

### Sample

We used data drawn from the wave 2 NESARC. The wave 1 NESARC (2001-2002) is a representative face-to-face survey that includes 43 093 adult residents of households or group quarters in the US, conducted by the National Institute on Alcoholism and Alcohol Abuse and described in detail elsewhere.^34,35^ The wave 2 survey (2004-2005) includes 86.7% of the original sample, corresponding to 34 653 completed interviews.^35^ The wave 2 NESARC data were weighted to be representative of the US civilian population based on the 2000 census.^35^ The research protocol, including written informed consent procedures, received full human subjects review and approval from the US Census Bureau and the Office of Management and Budget.^36^ The present study was conducted with this sample of 34 653 adults.

### Early Life Adversity

Early life adversity was modeled as a sum of *z* scores (scaled from 0.0 to 1.0) obtained on 53 items covering factors known to represent the general quality of the respondents’ early environment,^37,38^ between birth and age 18 years (eMethods 1 in Supplement 1).

### BPD

In the NESARC wave 2 interview, all participants were asked about lifetime BPD symptoms. These symptoms were assessed using the National Institute on Alcoholism and Alcohol Abuse Alcohol Use Disorder and Associated Disabilities Interview Schedule-IV, *DSM-IV* version.^39^ Analyses for the present study focused on the 9 *DSM-IV* BPD symptoms (eTable 1 in Supplement 1).^40^ In line with previous NESARC studies focusing on BPD, we only included symptoms causing social or occupational dysfunction.^41,42,43^

### Reproduction/Maintenance Trade-off

The trade-off meant to arbitrate the allocation of resources between short-term reproduction and somatic maintenance was modeled as a latent factor aiming at capturing the shared variance of 7 indicators commonly reported in human life history research.^38,44,45,46,47^ The reproductive indicators included the respondents’ number of children, the number of marriages, their age at first sexual intercourse, and history of sexually transmitted disease. The somatic maintenance indicators included the respondents’ body mass index at the time of the interview, their perceived physical health, and their metabolic risk factor (eMethods 2 in Supplement 1). A confirmatory factor analysis confirmed that our reproduction/maintenance trade-off latent factor correlated positively with the participants’ reproductive goals and negatively with their somatic maintenance traits (eFigure 1, eTable 2, and eTable 3 in Supplement 1). Higher scores on the latent factor positively correlated with immediate reproduction and negatively correlated with self-reported health status.

### Covariates

All models were adjusted for sex, age, and race (White vs non-White [Black, American Indian/Alaska Native, Asian/Native Hawaiian/Other Pacific Islander, or Hispanic]). Race was categorized as White vs non-White because members of minority racial and ethnic groups (ie, non-White) may experience an overall higher level of environmental adversity, which is a potential confounding factor in our models.^75,76,77^ All participants were asked to describe their race by selecting 1 or more categories defined by the investigator.

### Descriptive Statistics

We calculated the means for continuous variables, the proportions for binary variables, and standard errors among participants with BPD, participants without BPD, and in the full sample. All summary statistics and tests for between-group comparison of each variable took into account the sampling weights and design effects of the NESARC. Descriptive analyses were conducted using the *survey* R package^48^ with R software version 3.6.1 (R Foundation) and are summarized in the Table and eTables 2 and 4 in Supplement 1.

**Table.** Summary Statistics for the National Epidemiological Survey on Alcohol and Related Conditions Variables of Interest^a^

Characteristic	Total (N = 30 149)		No BPD (n = 29 257)		BPD (n = 892)		*P* value	Cohen *d*
	Mean (SE)	Median (IQR) [range]	Mean (SE)	Median (IQR) [range]	Mean (SE)	Median (IQR) [range]		
Female, % (SE)	52 (3.72 × 10^−5^)	NA	52 (3.77 × 10^−5^)	NA	57 (2.23 × 10^−4^)	NA	<.001	0.10
Male, % (SE)	48 (5.40 × 10^−5^)	NA	48 (5.42 × 10^−5^)	NA	43 (3.40 × 10^−4^)	NA	<.001	0.10
Age, y	47.78 (0.06)	46.00 (34.00-59.00) [20.00-90.00]	47.98 (0.06)	46.00 (34.00-59.00) [20.00-90.00]	40.54 (0.28)	40.00 (29.00-50.00) [20.00-90.00]	<.001	0.44
Race and ethnicity, % (SE)								
American Indian/Alaska Native	2.2 (7.36 × 10^−5^)	NA	2.2 (7.46 × 10^−5^)	NA	3.8 (4.42 × 10^−4^)	NA	<.001	0.11
Asian/Native Hawaiian/Other Pacific Islander	4.0 (7.29 × 10^−5^)	NA	4.1 (7.39 × 10^−5^)	NA	1.8 (4.5 × 10^−4^)	NA	<.001	0.12
Black	11 (7.01 × 10^−5^)	NA	11 (7.11 × 10^−5^)	NA	14 (4.2 × 10^−4^)	NA	<.001	0.10
Hispanic	12 (7.0 × 10^−5^)	NA	12 (7.09 × 10^−5^)	NA	11 (4.3 × 10^−4^)	NA	.003	0.03
White	71 (3.37 × 10^−5^)	NA	71 (3.42 × 10^−5^)	NA	70 (2.07 × 10^−4^)	NA	.15	NA
Age at first sexual intercourse, y	18.26 (0.01)	18.00 (16.00-20.00) [6.00-78.00]	18.33 (0.01)	18.00 (16.00-20.00) [6.00-78.00]	16.04 (0.08)	16.00 (14.00-18.00) [6.00-45.00]	<.001	0.60
No. of children	2.14 (8.60 × 10^−3^)	2.00 (1.00-3.00) [0-15.00]	2.14 (8.65 × 10^−3^)	2.00 (1.00-3.00) [0-15.00]	2.06 (0.06)	2.00 (0-3.00) [0-15.00]	<.001	0.04
No. of marriages	1.08 (3.40 × 10^−3^)	1.00 (1.00-1.00) [0-14.00]	1.08 (3.42 × 10^−3^)	1.00 (1.00-1.00) [0-14.00]	1.17 (0.02)	1.00 (0-2.00) [0-8.00]	.60	NA
History of sexually transmitted disease, % (SE)	0.6 (5.62 × 10^−6^)	NA	0.5 (5.26 × 10^−6^)	NA	3.8 (8.64 × 10^−5^)	NA	<.001	0.43
Perceived physical health	50.51 (0.04)	54.50 (47.30-57.50) [4.30-74.30]	50.58 (0.04)	54.50 (47.56-57.50) [4.30-74.30]	47.96 (0.20)	51.65 (39.78-57.90) [13.30-70.00]	<.001	0.25
Body mass index^b^	27.61 (0.02)	26.61 (23.62-30.54) [8.86-87.43]	27.59 (0.02)	26.58 (23.62-30.52) [8.86-87.43]	28.31 (0.14)	27.18 (23.49-31.96) [12.21-55.13]	<.001	0.12
Metabolic risk factors	0.81 (3.60 × 10^−3^)	1.00 (0-1.00) [0-4.00]	0.80 (3.55 × 10^−3^)	1.00 (0-1.00) [0-4.00]	0.96 (0.02)	1.00 (0-2.00) [0-4.00]	<.001	0.16
Early life adversity	0.08 (3 × 10^−4^)	0.04 (0.02-0.10) [0-1.00]	0.07 (3.01 × 10^−4^)	0.04 (0.02-0.10) [0-1.00]	0.20 (3.45 × 10^−3^)	0.16 (0.08-0.27) [0-0.91]	<.001	1.40

Abbreviations: BPD, borderline personality disorder; NA, not applicable.

^a^ χ^2^ Test with Rao-Scott second-order correction and Wilcoxon rank-sum test for complex survey samples was used.

^b^ Body mass index was calculated as weight in kilograms divided by height in meters squared.

### Structural Equation Models 

Structural equation models were used as our main multivariate analysis method. This analysis was conducted using Mplus version 8.1 (Muthén & Muthén).^49^ Model parameters estimation was conducted using mean- and variance-adjusted weighted least squares estimator.^49^ We then examined measures of goodness of fit, including the root mean squared error of approximation, the comparative fit index, the Tucker-Lewis index, and the standardized root mean square residual statistics. Root mean square error of approximation values less than 0.05, comparative fit index and Tucker-Lewis index values more than 0.95, and standardized root mean square residual values less than 0.08, which are commonly used to indicate good model fit, were used as cutoffs.

We evaluated a latent mediation model in which (1) the reproduction-maintenance trade-off latent factor was regressed on the early life adversity variable; (2) the BPD diagnostic was regressed on both the reproduction-maintenance trade-off latent factor; and (3) the early life adversity variable. Evidence for mediation of the link between adversity in early life and BPD diagnosis in adulthood by the latent reproductive/maintenance trade-off factor was assessed by 3 complementary analyses (eMethods 3 in Supplement 1). Analysis took place between August 2020 and June 2021.

#### Model Variations Across Sex

We further evaluated variation of the latent mediation model as well as the variation in the size of its estimates between male and female individuals. For this analysis, we compared nested models and tested differences between them with a robust χ^2^ difference for mean- and variance-adjusted weighted least squares estimators (eMethods 4 in Supplement 1).^50^

#### Sensitivity Analyses and *k*-Fold Cross-Validation

Several sensitivity analyses were conducted to examine the robustness of the results (eMethods 5 in Supplement 1). Risks of overfitting were estimated by applying a 10-fold cross-validation procedure on each model (eMethods 6 in Supplement 1).^51,52,53^

## Results

Analyses were run on a sample of 30 149 (87% of the initial sample) obtained after listwise deletion of participants with missing variables. The Table provides details of the summary statistics of each variable of interest, as well as the test of their difference between the group of participants with a *DSM-IV* BPD diagnosis (892 [3%]) and the group of participants without BPD (29 257 [97%]). Mean early life adversity, metabolic disorder score, and body mass index were significantly higher among participants with a diagnosis of BPD. The percentage of female individuals and the percentage of history of sexually transmitted disease in the year prior to the interview were also higher in the BPD subsample. Conversely, mean age at the time of the interview, age at first sexual intercourse, and physical health were all significantly lower among participants with BPD. The mean number of children was also significantly lower among participants with BPD. However, when age was adjusted for in a log-linear regression with number of children as the dependent variable, subsample (BPD vs non-BPD) as the predictor, and age of respondent as the covariate, individuals with BPD reported having significantly more children compared with those without BPD (b = 0.06; SE, 0.01; *t* = 4.09; *P* < .001; the b coefficient corresponding to a 6% difference between the 2 groups). Number of marriages and percentage of White participants did not significantly differ between the 2 groups. Model fit indices all indicated an excellent fit (comparative fit index, 0.990; Tucker-Lewis index, 0.973; root mean square error of approximation, 0.019 [95% CI, 0.017-0.021]; standardized root mean square residual, 0.030). The weighted percentages of categorical items and mean values of continuous items are provided in eTable 4 in Supplement 1. The observed correlation matrix can be found in eTable 5 in Supplement 1.

**Figure 1.**
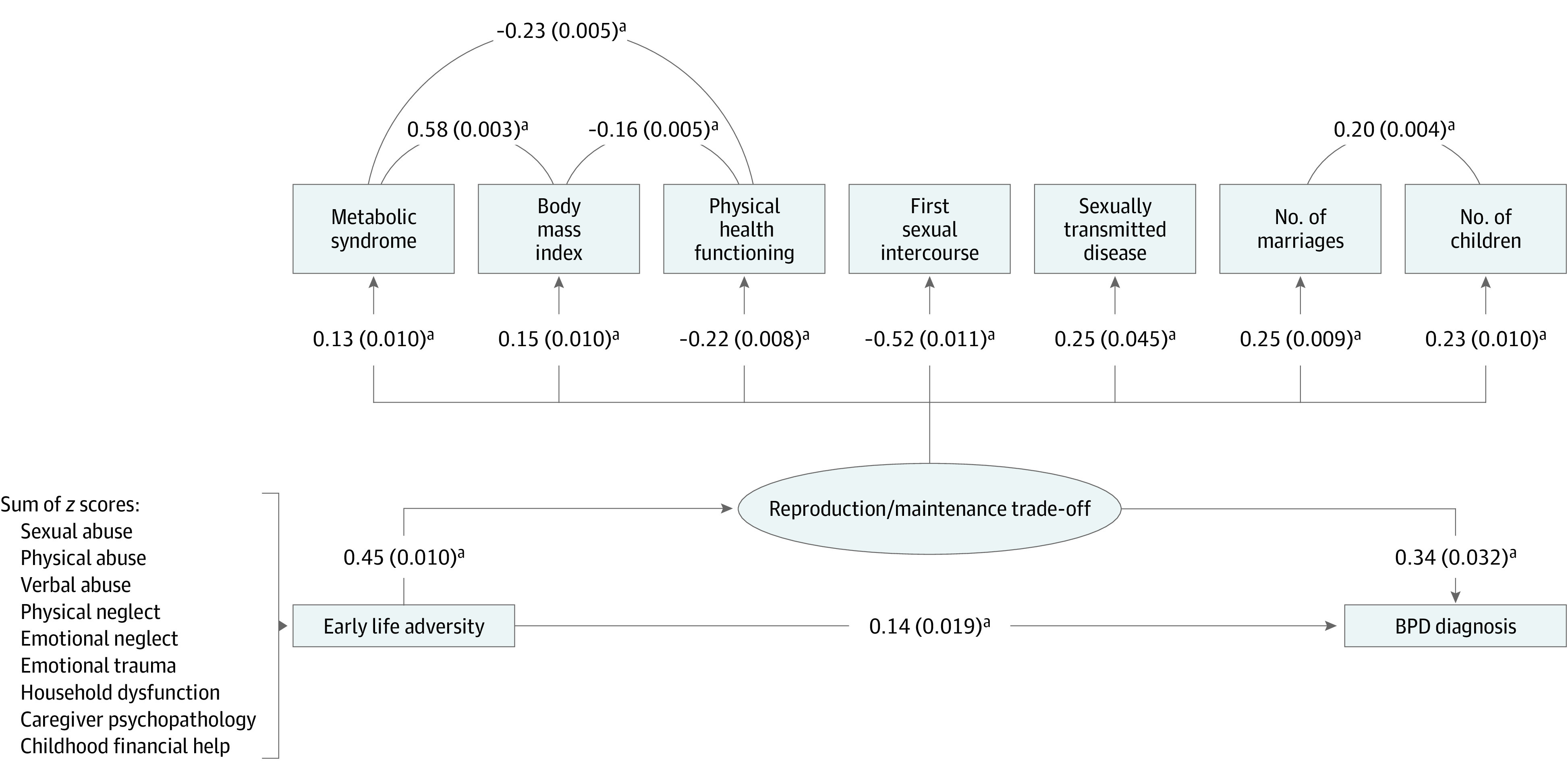
Main Latent Mediation Model This diagram describes the parameters of the model estimating the direct and indirect associations of early life adversity with the presence or absence of a borderline personality disorder (BPD) diagnosis. The ellipse represents the latent variable; rectangles represent its indicators. Early life adversity is modeled as a single composite variable, represented here by a rectangle. Paths between early life adversity, the reproduction/maintenance trade-off latent factor, and BPD diagnosis represent regressions. Paths between indicators and the reproduction/maintenance trade-off latent factor represent factor loadings. ^a^*P* < .001.

After adjusting for sex, age, and race, the model’s results indicated that (1) the early life adversity score was associated with the reproduction/maintenance trade-off latent factor score (standardized b = 0.448; SE, 0.010; *P* < .001); (2) the reproduction/maintenance trade-off latent factor score was positively associated with the occurrence of BPD diagnosis (standardized b = 0.335; SE, 0.032; *P* < .001), and so was (3) the early life adversity score (standardized b = 0.136; SE, 0.019; *P* < .001) (Figure 1 and eFigure 2 in Supplement 1).

These results indicate mediation of the association between adversity in early life and BPD diagnosis in adulthood by a latent reproductive-maintenance trade-off factor. Indeed, the association between early life adversity and BPD in the latent mediation model (b = 0.136; SE, 0.019; *P* < .001; Figure 1) was reduced in magnitude, relative to the same association in a simple probit regression model that did not include the reproduction/maintenance trade-off latent mediator (b = 0.284; SE, 0.011; *P* < .001).

**Figure 2.**
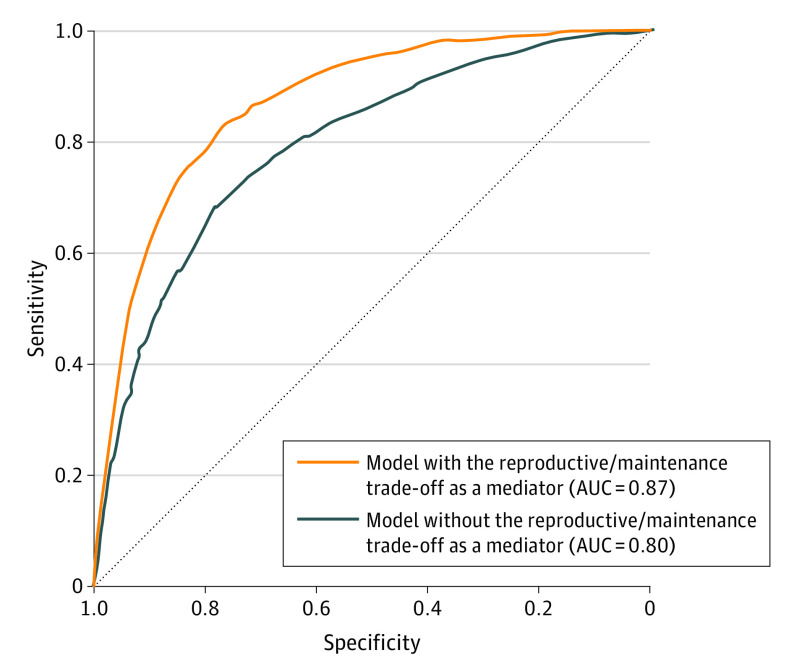
Classification Performance of the Main Latent Mediation Model The orange receiver operating characteristic curve represents the classification performance of the latent mediator model. The blue receiver operating characteristic curve represents the classification performance of a simple regression of the BPD borderline personality disorder variable on the early life adversity variable, which did not include the latent mediator of the reproduction/maintenance trade-off. For sensitivity, borderline personality disorder was present when present. For specificity, borderline personality disorder was absent when absent. AUC indicates area under the curve.

The validity of the latent mediator model was further supported by an analysis of its capacity to accurately indicate the presence or absence of BPD diagnosis (eMethods 3 in Supplement 1). The classification performance of this model was indeed higher than the classification performance of a simple probit regression (area under the receiver operating characteristic of the latent mediator model = 0.87; area under the receiver operating characteristic of probit regression = 0.80; Figure 2).

**Figure 3.**
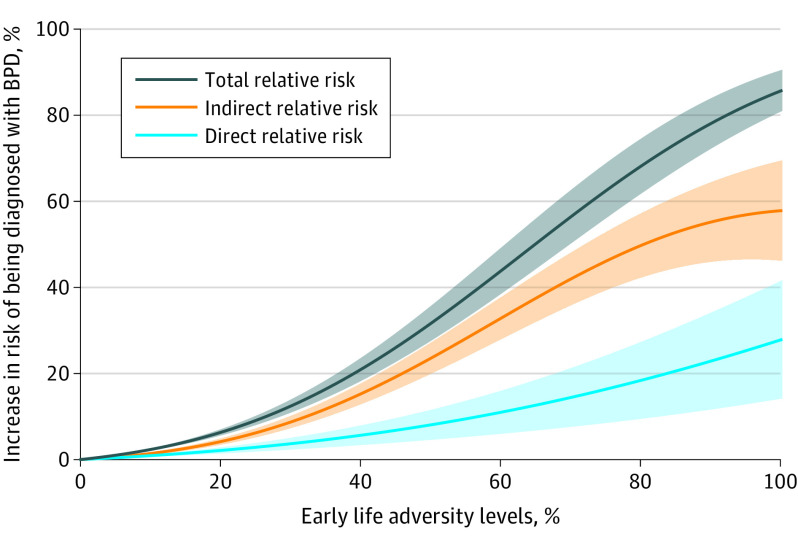
Percent Increase in Risk of Having a Diagnosis of Borderline Personality Disorder (BPD) Percent increase in risk of having a diagnosis of BPD due to indirect, direct, and total associations between early life adversity and the presence or absence of a BPD diagnosis, for all levels of early life adversity. The orange line and its shaded 95% CI indicates indirect relative risk^54^ between early life adversity and the presence or absence of a BPD diagnosis through the latent factor of the reproduction/maintenance trade-off, defined as the difference between the probability of having a diagnosis of BPD if one changes the latent factor score in the presence of a lower level of early life adversity, and the latent factor score expected if there had been an increase in early life adversity from its lower to its higher level, while keeping the value of the early life adversity variable fixed (at its upper bound). The light blue line and its shaded 95% CI indicates direct relative risk^54^ between early life adversity and the presence or absence of a BPD diagnosis, defined as the difference between the probability of having a diagnosis of BPD if the level of early life adversity is changed from its lower to its higher level, while keeping the latent factor score of the reproduction/maintenance trade-off at its expected value in the presence of a lower level of early life adversity. The dark blue line and its shaded 95% CI indicates the total relative risk between early life adversity and the presence or absence of a BPD diagnosis, defined as the sum of the direct and indirect relative risk.^54^ The median early life adversity level is taken as the baseline level (0).

Finally, we conducted an analysis that quantified the extent to which the risk of being diagnosed with BPD in adulthood after experiencing the highest levels of adversity in childhood (eMethods 3 in Supplement 1) could be modulated by individuals’ life strategy.^54^ This analysis showed that having experienced the highest level of adversity in childhood increased the risk of being diagnosed with BPD by 26.8% (direct relative risk = 0.268; SE, 0.067; *P* < .001). The latent trade-off factor, indicative of a strategy leading to more effort in immediate reproduction and less effort in somatic maintenance, further increased this risk by 56.5% (indirect relative risk = 0.565; SE, 0.056; *P* < .001). This value represents 67.8% of the total increase in risk of being diagnosed with BPD after having experienced the highest level of adversity in childhood (Figure 3). The estimates were similar across sex groups, with the exception of minor variations that we detail in eTables 6 and 7 and eFigures 3, 4, and 5 in Supplement 1.

Sensitivity analyses confirmed that the mediation of the reproduction/maintenance trade-off latent factor remained significant. Notably, it remained significant after adjusting for all lifetime axis I and II disorders (eFigures 6, 7, 8, and 9 in Supplement 1).

The 10-fold cross-validation revealed that the latent mediation model provided both very good fit indices and classification performance across training and test subsamples (eTable 8 and eFigure 10 in Supplement 1).

## Discussion

The objectives of this study were to test the existence of a latent factor by which seemingly disparate somatic traits and reproductive behaviors could follow a biologically plausible logic of organization and to examine how this latent factor is associated with early life adversity on the one hand and to the occurrence of BPD at adulthood on the other hand. To our knowledge, no prior theory explains the highly prevalent co-occurrence, in the BPD population (in comparison with population with other disorders, see^15,16^), of short-term reproductive behaviors and somatic comorbidities. Therefore, we sought to usefully exploit the concept of a trade-off between somatic maintenance and reproduction, predicted by life history theory, to account for the ecological origins of these distinct phenotypic expressions often reported independently in the BPD literature.

The results of our main SEM (Figure 1) support our initial hypothesis, showing that (1) respondents who scored high on the latent trade-off factor, ie, those who pursued immediate reproductive goals and who reported poorer somatic maintenance and health, were more likely to be diagnosed with BPD; (2) experiencing conditions of high adversity is associated with higher scores on the latent trade-off factor and an increased risk of meeting criteria for BPD in adulthood; and (3) the higher the respondents’ score on the latent trade-off factor, the more early life adversity is associated with BPD. An analysis of the latter association further shows that, for respondents who experienced high levels of adversity in early life, scoring high on the latent trade-off factor is associated with increased risk of having a diagnosis of BPD by 56.5% (Figure 3). This pattern of associations was observed in both male and female individuals, with only small differences between the 2 sexes (eMethods 4 and eFigure 5 in Supplement 1). Finally, cross-validation of the main SEM model highlights its ability to generalize its predictions to out-of-sample data, which confirms its robustness (Figure 2).

These results extend those of Otto and colleagues,^32^ who also examined associations between childhood adversity, somatic traits, and sociosexual preferences in patients with BPD. Our results support a view of BPD as the psychobehavioral expression of a broader coping strategy whereby individuals compensate for the adaptive costs of adverse life conditions by prioritizing a phenotype that provides rapid reproductive benefits at the expense of longer-term health and survival. According to this view, core components of borderline personality, eg, impulsivity, risk-taking, or negative emotionality, function to facilitate earlier sexuality, sexual promiscuity, and intrasexual competition for status and partners. Depressive symptoms and suicidal behaviors may also contribute to the construction of social support networks by eliciting empathy from others, thereby increasing the individual’s value as a socially desirable partner.^55^

The idea that BPD confers an early advantage in terms of reproductive success seems challenged by the statistics comparing the mean number of children of the BPD and non-BPD subsamples; in the former, individuals reported on average fewer children than in the latter (Table). However, this reproductive difference is only apparent and is explained by the 7.4-year difference in the mean age of the 2 groups, with the mean age of individuals with BPD of 40.5 years compared with 48 years for those without BPD. This large age difference biases the length of the reproductive window against the BPD sample (particularly for males who are not subject to menopause) and therefore likely inflated the mean fertility age of the sample without BPD. Indeed, when age is adjusted for in a log-linear regression with number of children as the dependent variable, subsample (BPD vs non-BPD) as the predictor, and age of respondent as the covariate, individuals with BPD reported having significantly more children than those without BPD. Note that all our SEM models are adjusted for age.

Our results neither invalidate nor minimize the value of alternative models emphasizing the influence of other proximal mechanisms preceding BPD expression.^56,57,58,59^ For example, Linehan's theory assumes that BPD is a consequence of emotional dysregulation.^60^ Gunderson and colleagues^57^ suggest that BPD is a consequence of feelings of loneliness and threat of rejection. Overall, the impact of emotional regulation mechanisms on symptom expression and their sociosexual correlates is a particularly important object of study insofar as these mechanisms constitute accessible therapeutic targets. Future work should integrate the mechanisms uncovered in this study with prior theories. While some recent theoretical contributions already point in this direction,^55,61,62^ their empirical validation remains to be conducted. Our work assumes that the emergence of BPD is a developmental response to adverse early life experiences. Consistent with this view, the heritability of BPD, estimated at 46% in a recent study,^63^ is lower than the heritability of attention-deficit/hyperactivity disorder, anorexia nervosa, autism spectrum disorder, bipolar disorder, or schizophrenia.^64^ Furthermore, heritability models assume that gene and environment have additive effects on the phenotype and thus necessarily underestimate the contribution of gene-environment interactions.^65^ Life history approaches to personality disorders integrate genetic causation and suggest that early life gene-environment interactions^21^ may increase the likelihood of expressing a reproduction-oriented life strategy, thereby increasing the risk of BPD.^61^

### Limitations

These findings must be balanced with several limitations. A first limitation is that important proxies of reproductive maturity (eg, age at puberty), which are thought to index reproduction-oriented life strategies, are not available in the NESARC. Second, information regarding institutionalized (incarcerated or hospitalized) or younger individuals is missing from NESARC,^42^ and future studies will need to determine whether our results generalize to these vulnerable populations. Third, axis I and II disorders were assessed by lay interviewers rather than mental health professionals, which might have resulted in false positives or negatives.^16^ However, the similarities between the NESARC results (using the same BPD definition we used in this study) and other BPD studies support the validity of the NESARC BPD assessment.^2,42,43^ Fourth, our indicators were retrospective and/or self-reporting in nature, hence subject to social desirability biases.^66^ For instance, adults tend to underreport experiences of early adversity.^67,68^ However, concerns about systematic biases in retrospective reports are mitigated by other prospective studies.^69,70^ Finally, our use of a cumulative model of adversity is questionable. Indeed, a recent trend in developmental psychopathology research emphasizes the predictive value of dimensional models.^71,72^

Despite its limitations, our study joins a growing body of work promoting a change in the functional status classically attributed to BDP.^25,30,31,32,61^ Whereas the common model of medical research views BPD and its multiple physiological and behavioral correlates as dysfunctions, evolutionarily informed approaches suggest that BPD-related traits offer advantages, not in terms of health but in terms of biological fitness, for navigating adverse environments where the risks of dying young or living in poor health conditions are higher.^21,30,73^

## Conclusions

Together, this work opens up promising avenues for improving the prevention and the management of this disorder. First, it highlights the importance of taking somatic and reproductive traits into account in clinical management. In practical terms, informing patients with BPD about the consequences of short-term reproductive behaviors could prevent them from falling into many psychosocial pitfalls, eg, unwanted parenthood, unstable interpersonal relationships, and sexual trauma. Furthermore, if an investment in immediate reproduction leads to less investment in somatic maintenance and thus to health problems later in life,^74^ it is all the more important to seek reducing reproductive impulsivity. These observations underscore the importance of coordinating mental health services with reproductive and general health services.

## Notes

Supplement 1. eMethods 1. List of items and scoring methods of the variables included in the models eMethods 2. Confirmatory factor analysis (CFA) eMethods 3. Structural equation model (SEM) eMethods 4. Model and estimates variations across sex eMethods 5. Sensitivity analysis eMethods 6. 10-Fold cross validation eTable 1. Items included in the structural equation models (RS = Reverse Coding) eTable 2. Univariate higher-order moment descriptive statistics eTable 3. Confirmatory Factor Analysis: correlation matrix of the variables included eTable 4. Univariate higher-order moment descriptive statistics eTable 5. Latent mediator model: correlation matrix of the variables included eTable 6. Descriptive statistics: men vs. women, whole sample eTable 7. Descriptive statistics: men vs. women, BPD sample eTable 8. 10-Fold cross validation eFigure 1. Confirmatory Factor Analysis (CFA) of the reproduction/maintenance trade-off latent construct eFigure 2. Latent mediator model classification performance versus simple probit regression classification performance eFigure 3. Latent mediator model in the women sample eFigure 4. Latent mediator model in the men sample eFigure 5. Partial metric invariance between men and women eFigure 6. Exclusion of all respondents who have been forced to have sexual intercourse during their infancy eFigure 7. Inclusion of an additional adjustment for participants’ personal income in the previous year eFigure 8. Inclusion of an additional adjustment for all lifetime Axis I and Axis II disorders eFigure 9. Age limit below 14 years old for childhood adverse events eFigure 10. 10-Fold cross validation: Area Under the Curve (AUC) of the Receiver Operating Characteristic (ROC) curves across train and test subsamples eReferences

Supplement 2. Data sharing statement
