# Supplementary material for: Associations Between Early Life Adversity, Reproduction-Oriented Life Strategy, and Borderline Personality Disorder
Source: JAMA Psychiatry. 2023 Apr 26;80(6):558–66. doi: 10.1001/jamapsychiatry.2023.0694 (PMC10134045; doi:10.1001/jamapsychiatry.2023.0694)
Supplement: Supplement 1. — eMethods 1. List of items and scoring methods of the variables included in the models eMethods 2. Confirmatory factor analysis (CFA) eMethods 3. Structural equation model (SEM) eMethods 4. Model and estimates variations across sex eMethods 5. Sensitivity analysis eMethods 6. 10-Fold cross validation eTable 1. Items included in the structural equation models (RS = Reverse Coding) eTable 2. Univariate higher-order moment descriptive statistics eTable 3. Confirmatory Factor Analysis: correlation matrix of the variables included eTable 4. Univariate higher-order moment descriptive statistics eTable 5. Latent mediator model: correlation matrix of the variables included eTable 6. Descriptive statistics: men vs. women, whole sample eTable 7. Descriptive statistics: men vs. women, BPD sample eTable 8. 10-Fold cross validation eFigure 1. Confirmatory Factor Analysis (CFA) of the reproduction/maintenance trade-off latent construct eFigure 2. Latent mediator model classification performance versus simple probit regression classification performance eFigure 3. Latent mediator model in the women sample eFigure 4. Latent mediator model in the men sample eFigure 5. Partial metric invariance between men and women eFigure 6. Exclusion of all respondents who have been forced to have sexual intercourse during their infancy eFigure 7. Inclusion of an additional adjustment for participants’ personal income in the previous year eFigure 8. Inclusion of an additional adjustment for all lifetime Axis I and Axis II disorders eFigure 9. Age limit below 14 years old for childhood adverse events eFigure 10. 10-Fold cross validation: Area Under the Curve (AUC) of the Receiver Operating Characteristic (ROC) curves across train and test subsamples eReferences [file jamapsychiatry-e230694-s001.pdf]

Supplemental Online Content

Baptista A, Chambon V, Hoertel N, et al. Associations between early life adversity, reproduction-oriented life strategy, and borderline personality disorder. *JAMA Psychiatry*. Published online April 26, 2023. doi:10.1001/jamapsychiatry.2023.0694

eMethods 1. List of items and scoring methods of the variables included in the models

eMethods 2. Confirmatory factor analysis (CFA)

eMethods 3. Structural equation model (SEM)

eMethods 4. Model and estimates variations across sex

eMethods 5. Sensitivity analysis

eMethods 6. 10-Fold cross validation

eTable 1. Items included in the structural equation models (RS = Reverse Coding)

eTable 2. Univariate higher-order moment descriptive statistics

eTable 3. Confirmatory Factor Analysis: correlation matrix of the variables included

eTable 4. Univariate higher-order moment descriptive statistics

eTable 5. Latent mediator model: correlation matrix of the variables included

eTable 6. Descriptive statistics: men vs. women, whole sample

eTable 7. Descriptive statistics: men vs. women, BPD sample

eTable 8. 10-Fold cross validation

eFigure 1. Confirmatory Factor Analysis (CFA) of the reproduction/maintenance trade-off latent construct

eFigure 2. Latent mediator model classification performance versus simple probit regression classification performance

© 2023 Baptista A et al. *JAMA Psychiatry.*

eFigure 3. Latent mediator model in the women sample

eFigure 4. Latent mediator model in the men sample

eFigure 5. Partial metric invariance between men and women

eFigure 6. Exclusion of all respondents who have been forced to have sexual intercourse during their infancy

eFigure 7. Inclusion of an additional adjustment for participants' personal income in the previous year

eFigure 8. Inclusion of an additional adjustment for all lifetime Axis I and Axis II disorders

eFigure 9. Age limit below 14 years old for childhood adverse events

eFigure 10. 10-Fold cross validation: Area Under the Curve (AUC) of the Receiver Operating Characteristic (ROC) curves across train and test subsamples

eReferences

This supplemental material has been provided by the authors to give readers additional information about their work.

© 2023 Baptista A et al. *JAMA Psychiatry.*

eTable 1. Items included in the structural equation models (RS = Reverse Coding)

early life adversity	physical abuse	before age 18, how often did parent/caregiver push, grab, shove, slap or hit you
		before age 18, how often did parent/caregiver hit you so hard that you had marks or bruises or were injured
	emotional abuse	before age 18, how often did parent/caregiver swear, insult or say hurtful things to you
		before age 18, how often did parent/caregiver threaten to hit you or throw something at you
		before age 18, how often did parent/caregiver make you fear that you would be physically hurt or injured
	sexual abuse	before age 18, how often did adult/other person fondle/touch you in sexual way when you didn't want this/were to young to know what was happening
		before age 18, how often did adult/other person have you touch them in sexual way when you didn't want this/were to young to know what was happening
		before age 18, how often did adult/other person attempt sexual intercourse with you when you didn't want this/were to young to know what was happening
		before age 18, how often did adult/other person have sexual intercourse with you when you didn't want this/were to young to know what was happening
	physical neglect	before age 18, how often did parent/caregiver make you do chores that were too difficult or dangerous for someone your age
		how often did parent/caregiver leave you alone or unsupervised before 10 years old
		before age 18, how often did you go without things you needed because a parent/caregiver spent the money on themselves
		before age 18, how often did parent/caregiver make you go hungry or not prepare regular meals

© 2023 Baptista A et al. *JAMA Psychiatry.*

		before age 18, how often did parent/caregiver ignore/fail to get you treatment when you were sick
	emotional neglect	before age 18, felt there was someone in family that wanted me to be a success (RS)
		before age 18, felt there was someone in family who helped me feel that I was important or special (RS)
		before age 18, felt that my family was a source of strength and support (RS)
		before age 18, felt that i was part of a close-knit family (RS)
		before age 18, felt that someone in my family believed in me (RS)
	caregiver psychopathology	before age 18, parent/other adult living in home was problem drinker/alcoholic
		before age 18, parent/other adult living in home had similar problems with drugs
		before age 18, parent/other adult living in home went to jail/prison
		before age 18, parent/other adult living in home treated/hospitalized for mental illness
		before age 18, parent/other adult living in home attempted suicide
		before age 18, parent/other adult living in home committed suicide
	household dysfunction	before age 18, how often did your father/other adult male push, grab, slap or throw something at your mother
		before age 18, how often did your father/other adult male hit your mother with a fist or with something hard

© 2023 Baptista A et al. *JAMA Psychiatry.*

		before age 18, how often did your father/other adult male repeatedly hit your mother for at least a few minutes
		before age 18, how often did your father/other adult male threaten your mother with a knife/gun or use a knife/gun to hurt her
	Childhood financial help	before you were 18 years old, was there ever a time when your family received money from government assistance programs like welfare, food stamps, general assistance, aid to families with dependent children, or temporary assistance for needy families?
	traumatic events (before age 18)	ever in active military combat
		ever serve as peacekeeper/relief worker in war zone/other terrorized area
		ever an unarmed civilian in war/revolution/military coup
		ever a refugee
		ever in serious/life-threatening accident
		ever in serious fire, tornado, flood, earthquake or hurricane
		ever sexually assaulted, molested, raped or experienced unwanted sex
		ever physically attacked/beaten/injured by spouse or romantic partner
		ever physically attacked/beaten/injured by anyone else

© 2023 Baptista A et al. *JAMA Psychiatry.*

		ever kidnapped or held hostage or as a pow
		ever stalked by anyone
		ever mugged, held up or threatened with a weapon
		ever had someone close to you die in terrorist attack
		ever had someone close to you injured in terrorist attack?
		ever yourself injured in terrorist attack
		ever had someone close to you directly experience a terrorist attack (95.34% missing values - excluded from the analysis)
		ever yourself directly experience a terrorist attack
		ever yourself indirectly experience a terrorist attack, like watching on tv
		other than terrorist attack, ever see someone badly injured/killed or ever unexpectedly see a dead body
		other than terrorist attack, ever have someone close to you die unexpectedly
		ever have someone close to you experience any other serious/life-threatening illness, accident or injury

© 2023 Baptista A et al. *JAMA Psychiatry.*

		someone close to you ever have any other very stressful/traumatic experience
		ever yourself have any other very stressful/traumatic experience
reproduction/maintenance trade-off	reproduction	how old were you when you first had sex/sexual intercourse, or have you never had sexual intercourse
		number of children ever had (incorporating information from wave 1)
		number of marriages (not counting living with someone as if married; incorporating information from wave 1)
		had any other sexually transmitted disease or venereal disease in the py & did a doctor or health professional confirm the diagnosis
	somatic maintenance	body mass index (bmi = reported weight in kilograms/[reported height in centimeters]
		participants' perceived physical health assessed using the physical component summary scale of the widely used 12-item short-form health survey (sf-12)
		z-score of the sum of the following variables: obesity = bmi > 30 (yes/no); had high cholesterol in the previous year (yes/no); had high blood pressure or hypertension in the previous year (yes/no) ; had diabetes or sugar diabetes in the previous year (yes/no)
borderline personality disorder		9 DSM-IV BPD symptoms associated with significant distress or impairment (i.e. 'frantic efforts to avoid real/imagined abandonment'; 'unstable/intense interpersonal relationships'; 'identity disturbance'; 'impulsivity'; 'suicidal/self-mutilation behavior'; 'affective instability'; 'chronic feelings of emptiness'; 'inappropriate/intense anger'; 'stress-related paranoid ideation')
covariates		sex

© 2023 Baptista A et al. *JAMA Psychiatry.*

		age
		ethnicity (white vs. non-white)

© 2023 Baptista A et al. *JAMA Psychiatry.*

eMethods 1. List of items and scoring methods of the variables included in the models

Early life adversity. We conceptualized early life adversity as a sum of environmental risk factors not necessarily correlated with one another but that all contribute to the cumulative probability of being diagnosed BPD. This is at odds with previous research using data from the NESARC, which have generally focused on the single effect of childhood maltreatment on BPD diagnostic. For example, the death of a parent as a child may be independent from being the victim of maltreatment. Even though they are different in nature and occur independently, such factors could cumulatively contribute to the emergence of BPD at later ages [1]. In line with this cumulative approach, we summed individual z-scores (scaled from 0.0 to 1.0) obtained on 53 items covering 9 environmental risk factors widely known to contribute to early life adversity levels: *Sexual abuse, Physical abuse, Physical neglect, Verbal abuse, Emotional neglect, Household dysfunction, Caregiver psychopathology*, *Traumatic event*, and *Economic scarcity*.

*Sexual abuse, physical abuse, verbal abuse, physical neglect, emotional neglect***.** Participants responded to 19 items informing about their exposure to physical, sexual, and emotional abuse, as well as physical and emotional neglect [2] before the age of 18 (before the age of 10 for the variable "*how often did parent/caregiver leave you alone or unsupervised*"; see eTable 1). These questions were adapted from earlier empirically validated scales scales[3,4]. Response options ranged from *'never'* (1) to *'very often'* (5), except for emotional neglect, which ranged from *'never'* to *'always'* and was reverse coded.

Household dysfunction. Participants responded to 4 questions asking whether their male caregiver had ever done any of the following to their female caregiver, before they were 18 years old: *'pushed, grabbed, slapped or threw something at her'*; *'kicked, bit, hit with a fist, or hit her with something hard'*; *'repeatedly hit her for at least a few minutes'*; or *'threatened her with a knife/gun or use a knife/gun to hurt her'*. Response options ranged from *'never'* (1) to *'very often'* (5).

Caregiver psychopathology. Participants responded to 6 items asking whether before the age of 18, at least one of their caregiver *'had problems with alcohol' or 'had problems with drugs'*, *'went to jail or prison'*, *'was treated or hospitalized for a mental illness'*, *'attempted suicide'* or *'committed suicide'*. These questions elicited a binary response (*'Yes'* vs. *'No'*).

Traumatic event. Participants responded to 23 items about their experience of traumatic events encompassing exposure to war, natural disasters, sexual violence, physical violence, or terrorist attack (see eTable 1). Participants who responded *'yes'* to any of these items, and reported having experienced the corresponding event before age 18, were considered to have been exposed to a traumatic event during their childhood.

Economic scarcity. Having experienced economic scarcity during childhood was assessed using the following item: 'Before you were 18 years old, was there ever a time when your family received money from government assistance programs like welfare, food stamps, general assistance, Aid to Families with Dependent Children, or Temporary Assistance for Needy Families?'. This question elicited a binary response ('*Yes*' vs. '*No*').

© 2023 Baptista A et al. *JAMA Psychiatry.*

eMethods 2. Confirmatory factor analysis (CFA)

Reproduction/maintenance trade-off latent factor

Measurement model. The hypothetical trade-off meant to arbitrate the resource allocation pattern between reproduction and somatic maintenance was modelled as a latent factor aiming at capturing the common variance of 7 indicators commonly reported in the human Life History research [5–8], and listed in e Table 1. The participants' reproductive goals were probed by collecting information on the following items: *'number of children ever had'*; *'number of marriages (not counting living with someone as if married)'*; *'how old were you when you first had sex/sexual intercourse, or have you never had sexual intercourse'; 'history of sexually transmitted disease (confirmed by a doctor or health professional in the year prior the interview)'*. Number of children was self-reported, in response to the following question asked to participants: "“How many children have you ever had, including those who are not now living? Please include adopted or foster children and any stepchildren who may have lived with you”. Although imperfect, it is the most accurate approximation of the number of biological children available in the database, and thus the better indicator of the respondents' fertility. To compensate for this imperfection, a covariance term was introduced between the indicators 'number of marriages' and 'number of children', the former being, by purely mechanical effect, more likely to impact the latter with non-biological children than with biological children. Participants' somatic maintenance functions were approximated using the following items: *body mass index* (BMI = reported weight in kilograms/[reported height in centimeters]^2); participants' *perceived physical health* assessed using the physical component summary scale of the widely used 12-item Short-Form Health Survey (SF-12, see [9]). Their scores were normalized to have a standard deviation of 10 and mean of 50. Lower scores indicate more physical disability. The participants' somatic maintenance state was further assessed by collecting information about their metabolic risk factors. Based on available data, database, we approximated participants' metabolic functioning risk as the z-scored sum of the following items: (i) obesity (BMI > 30), (ii); self-reported past-year presence of high cholesterol, (iii) high blood pressure and (iv) diabetes, which had to be confirmed by a doctor or health professional. The descriptive statistics of the 7 indicators are provided in eTable 2 right below.

© 2023 Baptista A et al. *JAMA Psychiatry.*

eTable 2. Univariate higher-order moment descriptive statistics

Variable	Sample Size	Mean	Variance	Skewness	Kurtosis	Minimum	Maximum	% with Min	% with Max	Percentile 20%	Percentile 60%	Percentile 40%	Percentile 80%	Median
Number of children	30149	2.141	3.481	1.647	6.106	0.000	15.000	21.71%	0.15%	0.000	2.000	2.000	3.000	2.000
Number of marriages	30149	1.085	0.594	1.498	8.945	0.000	14.000	19.64%	0.00%	1.000	1.000	1.000	2.000	1.000
Age at 1st sexual intercourse	30149	18.265	14.505	1.717	12.306	6.000	78.000	0.43%	0.00%	16.000	18.000	17.000	20.000	18.000
Perceived Physical health	30149	50.509	108.363	-1.535	1.862	4.300	74.300	0.00%	0.00%	42.700	55.600	52.100	57.800	54.000
BMI	30149	27.606	33.789	1.292	3.746	8.857	87.428	0.00%	0.00%	23.013	28.277	25.679	32.008	26.688
Metabolic risk factors	30149	0.808	0.981	1.160	0.695	0.000	4.000	47.52%	1.89%	0.000	1.000	0.000	2.000	1.000
History of sexually transmitted disease %(SE)	30149	0.6%												

© 2023 Baptista A et al. *JAMA Psychiatry.*

Confirmatory factor analysis (CFA) was used to test whether a latent factor could adequately fit the expected structure of a trade-off involving the 4 reproductive goals indicators and the 3 somatic maintenance indicators mentioned above. In order to take into account correlation patterns across observed indicators, residual correlations were allowed. For example, we expected that participants who married more often are mechanically more likely to have more children, independently of the resource allocation trade-off latent factor. Similarly, we expected that participants who had higher number of metabolic risk factors and greater BMI would tend to declare a poorer physical health state, independently of that factor.

For identification purposes, the variance of the reproduction/maintenance trade-off latent factor was scaled to have a variance of 1 and its mean centered to 0. This analysis was conducted using Mplus Version 8.1 [10], and it was adjusted for the complex NESARC sampling design. Model parameters estimation was conducted using delta parameterization and the variance-adjusted weighted least squares (WLSMV) estimator which is appropriate for categorical and dichotomous observed variables and departures from normality [10]. We then examined measures of goodness-of-fit, including the root mean squared error of approximation (RMSEA), the comparative fit index (CFI), the Tucker–Lewis index (TLI), and the SRMR statistics. RMSEA values < 0.05, CFI and TLI values >0.95, and SRMR values < 0.08 are commonly used to indicate good model's fit, and were used as cutoffs.

The CFA provided an adequate fit (CFI = 0.996, TLI = 0.984, RMSEA = 0.016 [0.013 - 0.020], SRMR = 0.018). Standardized parameter estimates, standard errors and associated p-values of the associations of each item with the latent factor are reported in eFigure 1. The pattern of correlations across items are indicated in eFigure 1. In line with the idea that investment in reproduction negatively correlate with investments in somatic maintenance, the CFA showed that higher scores on the latent factor were indeed associated with a poorer maintenance status (higher BMI, greater number of metabolic risk factors, lower self-reported physical health status) and short-term reproductive concerns (higher prevalence of past-year history of sexually transmitted disease, lower age at first sexual intercourse, greater number of marriages, and higher number of children). The observed correlation matrix can be found in eTable 3.

© 2023 Baptista A et al. *JAMA Psychiatry.*

eFigure 1. Confirmatory Factor Analysis (CFA) of the reproduction/maintenance trade-off latent construct.

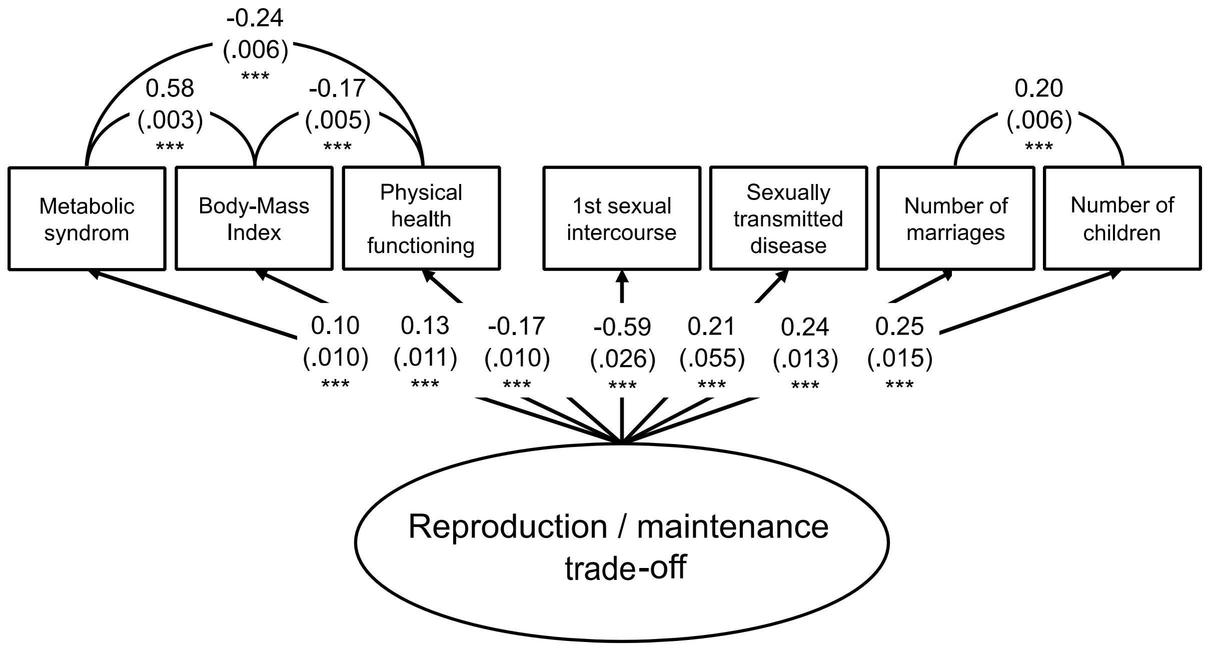

The ellipse represents the latent variable; rectangles represent its indicators. Paths between the indicators and the reproduction/maintenance trade-off latent factor represent factor loadings. Stars indicate significance: * for $p<0.05$, ** for $p<0.01$, and *** for $p<0.001$. CFI = 0.996, TLI = 0.984, RMSEA = 0.016 [0.013 - 0.020], SRMR = 0.018

© 2023 Baptista A et al. *JAMA Psychiatry.*

eTable 3. Confirmatory Factor Analysis: correlation matrix of the variables included

	number of children	number of marriages	age at 1st sexual intercourse	perceived physical health	bmi	history of sexually transmitted disease	Metabolic risk factors	
Number of children	1.000							
Number of marriages	0.254	1.000						
Age at 1st sexual intercourse	-0.159	-0.162	1.000					
Perceived Physical health	-0.044	-0.028	0.117	1.000				
BMI	0.080	0.031	-0.065	-0.193	1			
History of sexually transmitted disease	-0.006	0.060	-0.176	-0.114	-0.058	1.000		
Metabolic risk factors	0.043	0.023	-0.066	-0.257	0.585	0.015	1.000	

© 2023 Baptista A et al. *JAMA Psychiatry.*

eMethods 3. Structural equation model (SEM)

Mediation effect of the latent reproduction/maintenance trade-off in the association between early life adversity and BPD

The role of the reproduction/maintenance trade-off (as identified by the CFA described in the previous section) in mediating the effect of early life adversity on BPD was assessed via three complementary and widely accepted methods.

Causal steps. First, we evaluated whether the association between early life adversity and BPD estimated by a simple probit regression model changed in magnitude when the reproduction/maintenance trade-off was added as a latent mediator. A limitation of this approach is that it is qualitative, meaning that it lacks a formal significance test of the mediation effect [11].

Joint significance. Second, we evaluated the statistical significance of two estimated associations: (i) early life adversity and the reproduction/maintenance trade-off, (ii) the reproduction/maintenance trade-off and BPD diagnosis. If both are significant, there is evidence for a mediation effect [11].

Causally defined direct and indirect relative risks. The traditional *'product of coefficients'* is difficult to interpret when the outcome is dichotomous, as it is the case in the present study [12]. For this reason, we estimated the mediation effect of the reproduction/maintenance trade-off factor on BPD diagnosis using the more interpretable counterfactual definition of indirect causal effect on dichotomous outcome, implemented in Mplus version 8.1 [10]. Following this method, we first defined *upper adversity level* = the maximum level of adversity one could suffer from in the NESARC sample, and *lower adversity level* = the median level of adversity in the total NESARC sample. We then defined the direct relative risk of BPD diagnosis as follows:

$$\begin{gathered} Direct\ relative\ risk \\ = \\ Prob[(BPD = 1|(upper\ adversity\ level, reproduction/maintenance\ trade\ off(lower\ adversity\ level))] \\ - \\ Prob[(BPD = 1|(lower\ adversity\ level, reproduction/maintenance\ trade\ off(lower\ adversity\ level))] \end{gathered}$$

The expression should be read as the difference between the probability of *BPD* diagnosis at the *upper adversity level* and at the *lower adversity level*, when the value of the mediator *reproduction/maintenance trade-off* does not change and is fixed at its expected value in the presence of the *lower adversity level*.

We next defined the indirect relative risk of BPD diagnosis through the reproduction/maintenance trade-off latent factor as follows:

$$\begin{gathered} Indirect\ relative\ risk \\ = \\ Prob[(BPD = 1|(upper\ adversity\ level, reproduction/maintenance\ trade\ off(upper\ adversity\ level))] \\ - \\ Prob[(BPD = 1|(upper\ adversity\ level, reproduction/maintenance\ trade\ off(lower\ adversity\ level))] \end{gathered}$$

© 2023 Baptista A et al. *JAMA Psychiatry.*

The expression should be read as the difference between the probability of *BPD diagnosis* in the *upper adversity level* when the mediator *reproduction/maintenance trade off* changes from its value it would obtain at the *lower adversity level* to its value it would obtain at the *upper adversity level*.

These differences were calculated and tested against zero using standard errors obtained with the delta method [13].

eTable 4. Univariate higher-order moment descriptive statistics

Variable	Sample Size	Mean	Variance	Skewness	Kurtosis	Minimum	Maximum	% with Min	% with Max	Percentile 20%	Percentile 60%	Percentile 40%	Percentile 80%	Median
Number of children	30149	2.141	3.481	1.647	6.106	0.000	15.000	21.71%	0.15%	0.000	2.000	2.000	3.000	2.000
Number of marriages	30149	1.085	0.594	1.498	8.945	0.000	14.000	19.64%	0.00%	1.000	1.000	1.000	2.000	1.000
Age at 1st sexual intercourse	30149	18.265	14.505	1.717	12.306	6.000	78.000	0.43%	0.00%	16.000	18.000	17.000	20.000	18.000
Perceived Physical health	30149	50.509	108.363	-1.535	1.862	4.300	74.300	0.00%	0.00%	42.700	55.600	52.100	57.800	54.000
BMI	30149	27.606	33.789	1.292	3.746	8.857	87.428	0.00%	0.00%	23.013	28.277	25.679	32.008	26.688
Metabolic risk factors	30149	0.808	0.981	1.160	0.695	0.000	4.000	47.52%	1.89%	0.000	1.000	0.000	2.000	1.000
Childhood adversity	30149	0.077	0.009	2.701	10.312	0.000	1.000	12.08	0.00	0.014	0.062	0.033	0.124	0.046
Age	30149	47.780	284.285	0.409	-0.636	20.000	90.000	0.07%	0.62%	33.000	51.000	42.000	64.000	47.000
Female %(SE)	30149	51.7%												
White %(SE)	30149	71.1%												
History of sexually transmitted disease %(SE)	30149	0.6%												
BPD	30149	2.7%												

© 2023 Baptista A et al. *JAMA Psychiatry.*

eTable 5. Latent mediator model: correlation matrix of the variables included.

	bpd	number of children	number of marriages	age at 1st sexual intercourse	perceived physical health	bmi	history of sexually transmitted disease	metabolic risk factors	Age	Female	White	Childhood adversity
bpd	1.000											
number of children	0.019	1.000										
number of marriages	0.041	0.343	1.000									
age at 1st sexual intercourse	-0.299	-0.050	-0.067	1.000								
perceived physical health	-0.092	-0.196	-0.150	0.017	1.000							
bmi	0.049	0.081	0.025	-0.067	-0.182	1.000						
history of sexually transmitted disease	0.377	-0.116	-0.034	-0.206	-0.012	-0.084	1.000					
metabolic risk factors	0.063	0.182	0.136	0.019	-0.360	0.550	-0.099	1.000				
Age	-0.209	0.394	0.340	0.223	-0.380	0.008	-0.295	0.354	1.000			
Female	0.043	0.066	0.047	0.107	-0.060	-0.051	0.098	0.025	0.045	1.000		
White	-0.012	-0.045	0.132	0.052	0.002	-0.064	0.027	0.001	0.161	-0.008	1.000	
Childhood adversity	0.299	0.049	0.077	-0.240	-0.092	0.068	0.155	0.032	-0.098	0.029	-0.037	1.000

© 2023 Baptista A et al. *JAMA Psychiatry.*

eFigure 2. Latent mediator model classification performance versus simple probit regression classification performance

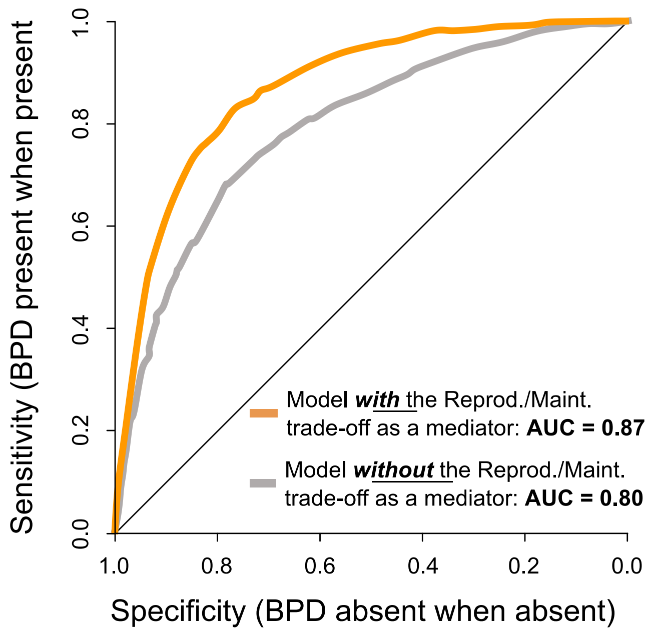

To further examine the model validity, we also assessed its performance in predicting the presence or absence of a BPD diagnosis in each participant. This classification performance was measured using the Area Under the ROC Curve (AUC) [14]. The ROC curve presents the true-positive rate (the proportion of observations belonging to class A and classified as A, i.e., hit rate) as a function of the false-positive rate (i.e., the proportion of observations belonging to class B and classified as A, i.e., false alarm rate). Importantly, AUC analysis provides an unbiased measure of classification accuracy, robust to imbalanced problems and independent of the statistical distribution of the classes. The AUC was derived from each participant's propensity score. Here the propensity score corresponded to the expected value of the SEM model's continuous latent response variable behind the BPD binary observed variable (BPD*), given the observed level of early life adversity and the corresponding resource allocation latent factor score [15].

eMethods 4. Model and estimates variations across sex

We also evaluated variation of the latent mediator model (configural invariance) as well as the variation in the size of its estimates (metric invariance) between men and women. For this, we compared nested models and tested differences between them with a robust chi-squared difference test suitable for WLSMV estimators [16].

© 2023 Baptista A et al. *JAMA Psychiatry.*

eTable 6. Descriptive statistics: men vs. women, whole sample

	Men (N = 12747)			Women (N = 17402)			Total (N = 30149)			p-value[a]
	Mean (SE)	Median (IQR)	Min - Max	Mean (SE)	Median (IQR)	Min - Max	Mean (SE)	Median (IQR)	Min - Max	
BPD%(SE)	2,4% (1.655e-05)			3,0% (1.759e-05)			2,7% (1.211e-05)			<0,001
Age	46,99 (0.0823)	45,00 (34,00-58,00)	20,00 - 90,00	48,51 (0.0863)	47,00 (35,00-60,00)	20,00 - 90,00	47,78 (0.062)	46,00 (34,00-59,00)	20,00 - 90,00	<0,001
White %(SE)	71% (4.834e-05)			71% (4.701e-05)			71% (3.37e-05)			0,019
Age at 1st sexual intercourse	17,84 (0.0187)	17,00 (16,00, 19,00)	6,00-73,00	18,66 (0.016)	18,00 (16,00-20,00)	6,00-78,00	18,26 (0.014)	18,00 (16,00-20,00)	6,00-78,00	<0,001
Number of children	2,01 (0.0103)	2,00 (0,00-3,00)	0,00-15,00	2,26 (0.011)	2,00 (1,00-3,00)	0,00-15,00	2,14 (0.009)	2,00 (1,00-3,00)	0,00-15,00	<0,001
Number of marriages	1,05 (0.005)	1,00 (1,00-1,00)	0,00-8,00	1,12 (0.004)	1,00 (1,00, 1,00)	0,00-14,00	1,08 (0.003)	1,00 (1,00-1,00)	0,00-14,00	<0,001
History of sexually transmitted disease%(SE)	0,4% (6.853e-06)			0,7% (8.770e-06)			0,6% (5.616e-06)			<0,001
Perceived Physical health	51,16 (0.067)	54,80 (48,80-57,20)	4,30-70,10	49,91 (0.050)	54,00 (45,50-57,50)	4,60-74,30	50,51 (0.043)	54,50 (47,30-57,50)	4,30-74,30	<0,001
BMI	27,91 (0.028)	27,13 (24,41-30,41)	12,21-71,74	27,32 (0.027)	25,85 (22,67-30,73)	8,86-87,43	27,61 (0.020)	26,61 (23,62-30,54)	8,86-87,43	<0,001
Metabolic risk factors	0,78 (0.005)	0,00 (0,00-1,00)	0,00-4,00	0,83 (0.004)	1,00 (0,00-1,00)	0,00-4,00	0,81 (0.004)	1,00 (0,00-1,00)	0,00-4,00	<0,001
early life adversity	0,07 (0.0004)	0,05 (0,02-0,10)	0,00-1,00	0,08 (0.0004)	0,04 (0,02-0,10)	0,00-0,99	0,08 (3e-04)	0,04 (0,02-0,10)	0,00-1,00	<0,001

[a] chi-squared test with Rao & Scott's second-order correction; Wilcoxon rank-sum test for complex survey samples

© 2023 Baptista A et al. *JAMA Psychiatry.*

eTable 7. Descriptive statistics: men vs. women, BPD sample

	Men (N = 335)			Women (N = 557)			Total (N = 892)			p-value[a]
	Mean (SE)	Median (IQR)	Min - Max	Mean (SE)	Median (IQR)	Min - Max	Mean (SE)	Median (IQR)	Min - Max	
Age	41.25 (0.377)	41.00 (28.00-51.00)	20.00-84.00	40.00 (0.415)	39.00 (30.00-48.00)	20,00 - 90,00	40.54 (0.278)	40.00 (29.00-50.00)	20,00 - 90,00	0.088
White %(SE)	67% (0.0003)			72% (0.0003)			70% (0.0002)			0.006
Age at 1st sexual intercourse	15.85 (0.098)	16.00 (13.98-18.00)	16.1939.00	18,66 (0.110)	16.00 (15.00-18.00)	6.00-45.00	16.04 (0.082)	16.00 (14.00-18.00)	6.00-45.00	0.026
Number of children	1.77 (0.048)	1.00 (0.00-3.00)	0.00-11.00	2.28 (0.098)	2.00 (1.00-3.00)	0.00-15.00	2.06 (0.064)	2.00 (0.00-3.00)	0,00-15,00	<0,001
Number of marriages	1.04 (0.027)	1.00 (0.00-2.00)	0,00-8,00	1.27 (0.026)	1.00 (1.00-2.00)	0.00-7.00	1.17 (0.021)	1.00 (0.00-2.00)	0.00-8.00	<0,001
History of sexually transmitted disease %(SE)	2.1% (9.792e-05)			5.1% (0.0001)			3.8% (8.638e-05)			0.004
Perceived Physical health	47.77(0.328)	50.68 (40.11-57.21)	16.30-70.00	48.10 (0.290)	52.20 (39.32-58.50)	13.30-68.60	47.96 (0.204)	51.65 (39.78-57.90)	13.30-70.00	0.067
BMI	27.93 (0.183)	27.09 (23.71-31.35)	12.21-53.22	28.60 (0.197)	27.28 (23.17-32.63)	16.06-55.13	28.31 (0.138)	27.18 (23.49-31.96)	12.21-55.13	0.4
Metabolic risk factors	1.02 (0.030)	1.00 (0.00-2.00)	0,00-4,00	0.92 (0.028)	1.00 (0.00-1.06)	0,00-4,00	0.96 (0.022)	1.00 (0.00-2.00)	0,00-4,00	0.003
early life adversity	0.18 (0.004)	0.14 (0.08-0.23)	0.00-0.77	0.21 (0.005)	0.17 (0.09-0.28)	0.00-0.91	0.20 (0.004)	0.16 (0.08-0.27)	0.00-0.91	<0,001

[a] chi-squared test with Rao & Scott's second-order correction; Wilcoxon rank-sum test for complex survey samples

© 2023 Baptista A et al. *JAMA Psychiatry.*

4.1. Configural invariance between men and women

To test the configural invariance of our latent mediator model between men and women, we compared nested SEM models and tested differences between them with a robust chi-squared difference test (labeled χ^2 in the reported results below) suitable for WLSMV estimators [16], using the DIFFTEST procedure [16].

First, a configural invariance model was specified, in which a single latent mediator model was estimated simultaneously in the men and women samples. Model fit indices all indicated that this model provided a good fit to the data (CFI = 0.983, TLI = 0.964, RMSEA = 0.025 [0.023 0.027]; SRMR = 0.042). This indicated that the latent mediator model has the same configuration across men and women groups [17] (see eFigure 3 and eFigure 4 below for details).

eFigure 3. Latent mediator model in the women sample

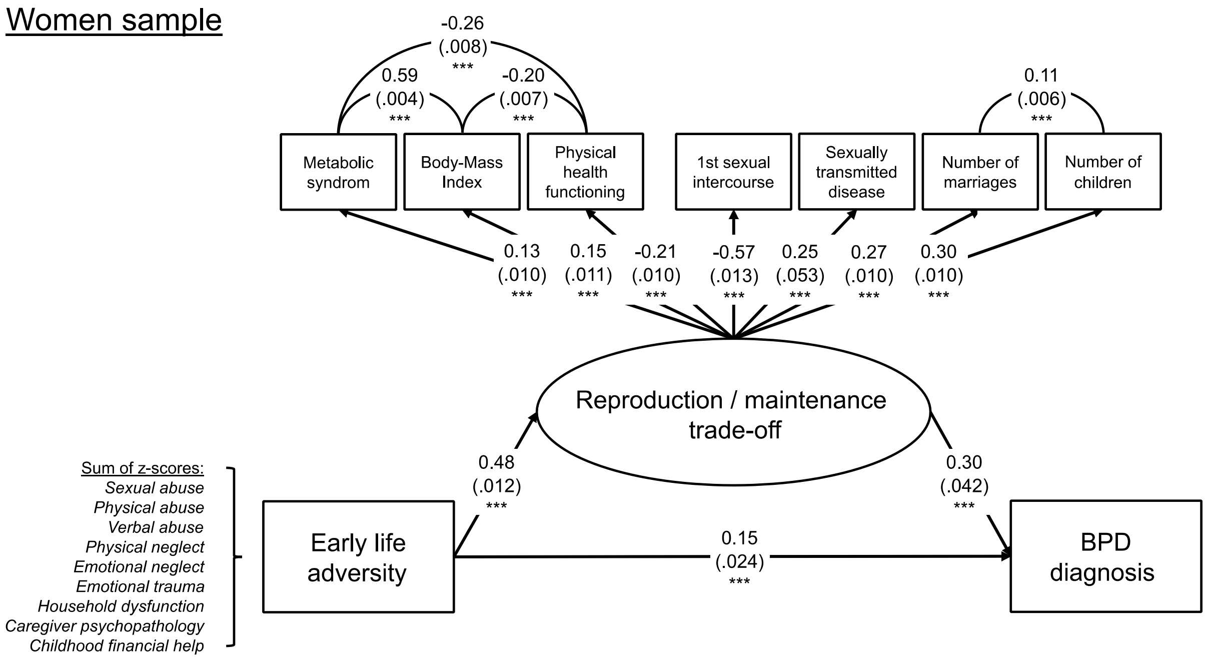

Latent Mediation Model for the effect of Early life adversity on the presence or absence of a BPD diagnosis in the women sample. The ellipse represents the latent variable; rectangles represent its indicators. Early life adversity is modeled as a single composite variable, here represented by a rectangle. Paths between Early life adversity, the reproduction/maintenance trade-off latent factor and BPD diagnosis represent regressions. Paths between the indicators and the reproduction/maintenance trade-off latent factor

© 2023 Baptista A et al. *JAMA Psychiatry.*

represent factor loadings. Stars indicate significance: * for p < 0.05, ** for p < 0.01, and *** for p < 0.001. CFI = 0.983; TL I= 0.964; RMSEA = 0.025 (0.023 – 0.027); SRMR = 0.042.

eFigure 4. Latent mediator model in the men sample

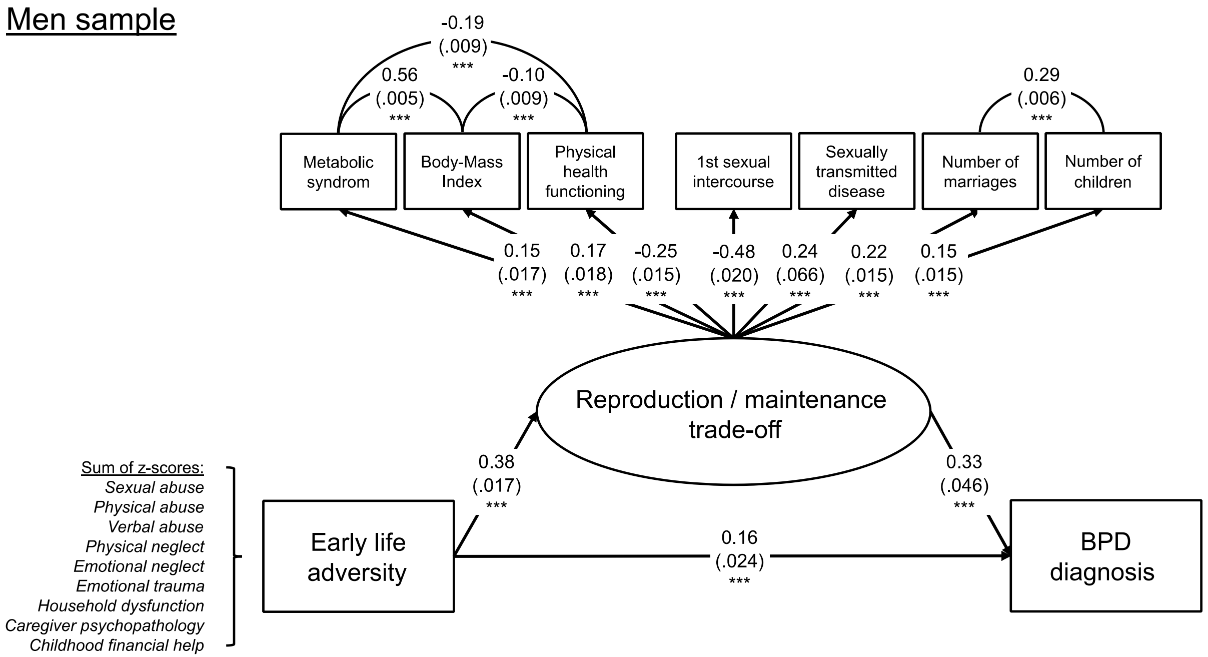

Latent Mediation Model for the effect of Early life adversity on the presence or absence of a BPD diagnosis in the men sample. The ellipse represents the latent variable; rectangles represent its indicators. Early life adversity is modeled as a single composite variable, here represented by a rectangle. Paths between early life adversity, the reproduction/maintenance trade-off latent factor and BPD diagnosis represent regressions. Paths between the indicators and the reproduction/maintenance trade-off latent factor represent factor loadings. Stars indicate significance: * for p < 0.05, ** for p < 0.01, and *** for p < 0.001. CFI = 0.983; TLI = 0.964; RMSEA = 0.025 (0.023 – 0.027); SRMR = 0.042.

© 2023 Baptista A et al. *JAMA Psychiatry.*

4.2. Partial metric invariance between men and women

However, configural invariance does not imply that men and women models show equivalent associations across early life adversity, the reproduction/maintenance trade-off latent factor, and BPD diagnosis on the one hand, and across the reproduction/maintenance trade-off latent factor with its observed indicators on the other hand. Therefore, we tested a more constrained version of metric invariance, which is met when the condition that the model estimates hold across sex groups (Vandenberg and Lance 2000). To this aim, we investigated the equality of the unstandardized estimates between men and women samples in a metric invariance model [18] by constraining estimates to be equal across sex groups. The comparison of the metric invariance model with the configural invariance model revealed that the former fitted the data significantly worse than the latter (χ^2 = 495.75, p < 0.001).

An examination of the modification indices revealed predominant misfits for the constrained correlation between the indicators ‘Number of marriages’ and ‘Number of children’, ‘BMI’ and ‘Metabolic risk factors', ‘BMI’ and ‘Perceived Physical health'; as well as predominant misfits for the constrained loadings of the indicators ‘Number of Children’ and ‘Age at 1st sexual intercourse’ on the reproduction/maintenance trade-off latent factor.

Importantly, freeing these correlations and factor loadings between the two sex samples (eFigure 5) did not change the result of the model comparison: the partial metric invariance model still fitted significantly worse than the configural invariance model (all $\chi^2 > 37.32$, all ps < 0.001).

Modification indices also indicated a predominant misfit for the constrained correlation between the indicators 'Metabolic risk factors' and ‘Perceived Physical health'. However, this time freeing this covariance term between the two sex samples (eFigure 5) did change the result of the model comparison: the partial metric invariance model and the configural invariance model fitted the data equally well (χ^2reproduction/maintenance trade-off = 17.434, p =0.10).

The fact that criterion for partial metric invariance (i.e., “weak invariance”) were met is an indication that the estimates were equivalent across groups or, more simply, that the same model was being measured in each group, with the exception of minor variations: 1) the estimated absolute values of the associations between ‘Age at first sexual intercourse’ as well as ‘Number of children’ with the reproduction/maintenance trade-off latent mediator were higher in women; 2) the correlations between BMI, 'Metabolic risk factors', and Perceived physical health were stronger in women; and 3) the correlations between number of children and number of marriages was lower in women.

© 2023 Baptista A et al. *JAMA Psychiatry.*

eFigure 5. Partial metric invariance between men and women

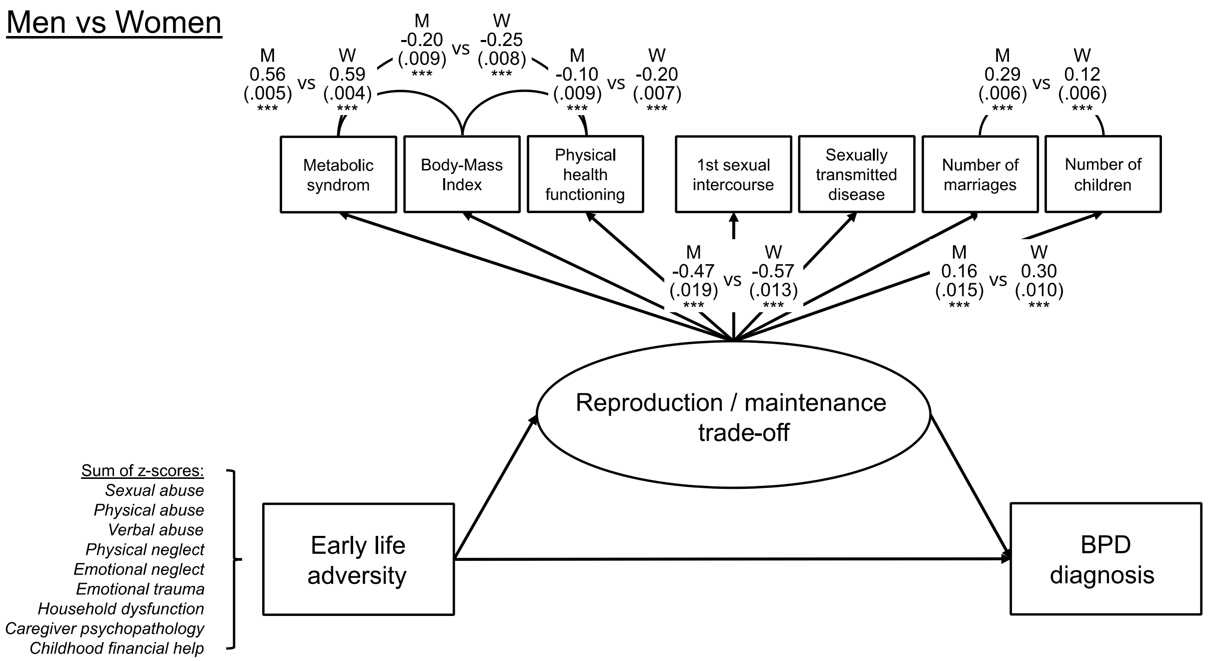

eMethods 5. Sensitivity analysis

We then submitted our main mediation model to a set of sensitivity analyses.

In a first replication of the model, we removed all respondents who reported having been forced to have sexual intercourse during their childhood. Indeed, participants were asked to report their age at first sexual intercourse, regardless of whether it was consented or not. This could have artificially increased the association between early life adversity and the reproduction/maintenance trade-off factor.

Second, we replicated the model after having included an additional adjustment for participants' personal income in the previous year. The aim was to ensure that the associations persisted even after adjusting for current level of economic adversity.

In the third replication, we included an additional adjustment for lifetime Axis I mood and anxiety disorders. By doing so, we aimed to reduce the risk that psychiatric comorbidities frequently observed in BPD patients affected the direct and indirect effects of the early life adversity and the reproduction/maintenance trade-off constructs on the BPD diagnosis [19].

© 2023 Baptista A et al. *JAMA Psychiatry.*

Finally, in the fourth and last replication of the mediation model, we restricted early life adversity to all adverse events experienced by participants before age 14 (i.e. the 23 items related to traumatic events; see eTable 1) instead of age 18. Here, the goal was to check whether the measurement and structural properties of the mediational model were kept even after narrowing adverse experiences to the developmental period comprised between conception and sexual maturity.

The causally defined indirect effect of early life adversity on BPD diagnosis through the reproduction maintenance trade-off latent was estimated in each the four sensitivity analyses described above. Several interesting results emerged.

Firstly, removing respondents who have been forced to have sexual intercourse during their infancy did not cancel out the indirect effect of early life adversity on BPD diagnosis ($p < .001$), even though this effect decreased in size (eFigure 6). Indeed, in this 1st sensitivity analysis the indirect effect corresponded to a 38.5±7.5% increase in the risk of being diagnosed BPD (41.12% of the total effect), while such risk was estimated at 56.5% in the original model.

Secondly, adjusting for participants' personal current income also led to a marginal decrease in the size of the indirect effect but, as in the previous analysis, did not impact its significance ($p < .001$) (eFigure 7). In this 2nd sensitivity analysis, the indirect effect corresponded to a 51±5.7% increase in the risk of being diagnosed BPD (62.89% of the total effect), which is slightly lower than the 56.5% risk estimated in the original model.

Thirdly, the indirect effect remained significant even after setting the period of adversity exposure below age 14 (eFigure 9). It corresponded to a 46.3±7.5% increase in the risk of having a diagnosis of BPD ($p < .001$) (48.94% of the total effect).

Fourthly, the indirect effect remained significant even after adjusting for all lifetime Axis I and Axis II disorders (eFigure 8) included in the database (i.e. lifetime psychotic illness or episode, mood disorders, anxiety disorders, ADHD, conduct disorder, substance use disorders, all personality disorders), with a 2.4±1.1% increase in the risk of being diagnosed of BPD ($p = .022$). Notably, this indirect effect represented 100% of the total effect. Indeed, our results did not provide evidence for a direct causal effect of early life adversity on BPD diagnosis ($p = .95$).

In conclusion, results of the sensitivity analyses support the robustness of our original model.

© 2023 Baptista A et al. *JAMA Psychiatry.*

eFigure 6. Exclusion of all respondents who have been forced to have sexual intercourse during their infancy

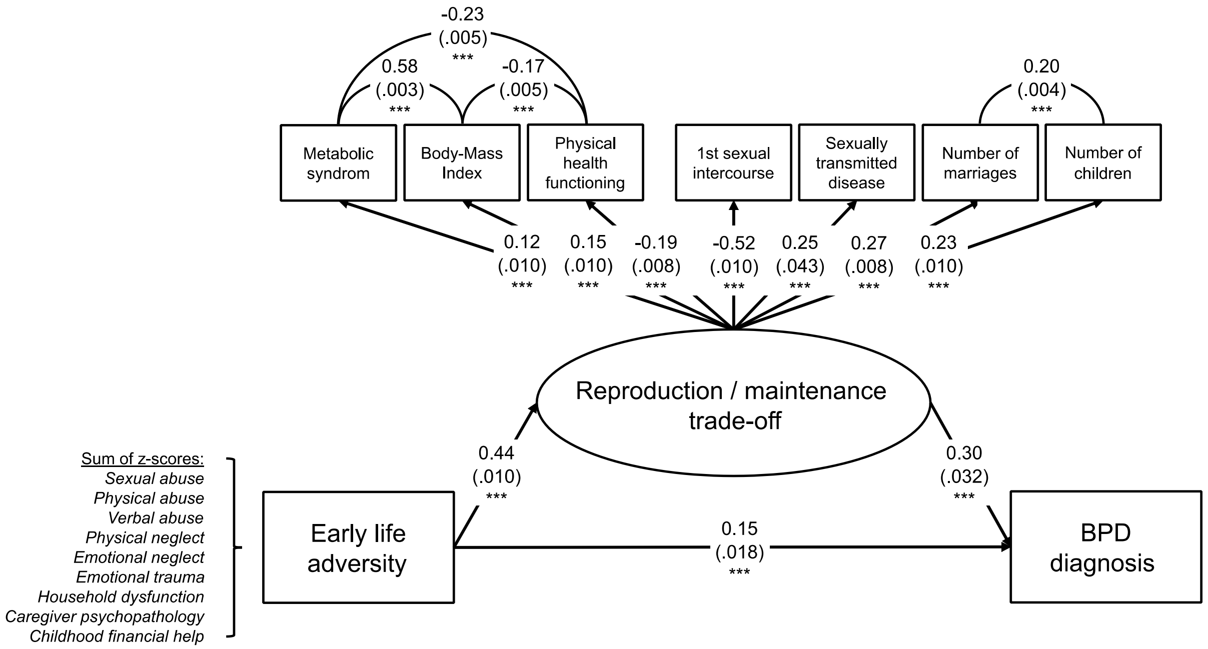

Latent Mediation Model for the effect of Early life adversity on the presence or absence of a BPD diagnosis. The ellipse represents the latent variable; rectangles represent its indicators. Early life adversity is modeled as a single composite variable, here represented by a rectangle. Paths between Early life adversity, the reproduction/maintenance trade-off latent factor and BPD diagnosis represent regressions. Paths between the indicators and the reproduction/maintenance trade-off latent factor represent factor loadings. Stars indicate significance: * for p<0.05, ** for p<0.01, and *** for p<0.001. CFI= 0.988; TLI= 0.968; RMSEA= 0.020 (0.018 -0.022); SRMR= 0.032

© 2023 Baptista A et al. *JAMA Psychiatry.*

eFigure 7. Inclusion of an additional adjustment for participants' personal income in the previous year

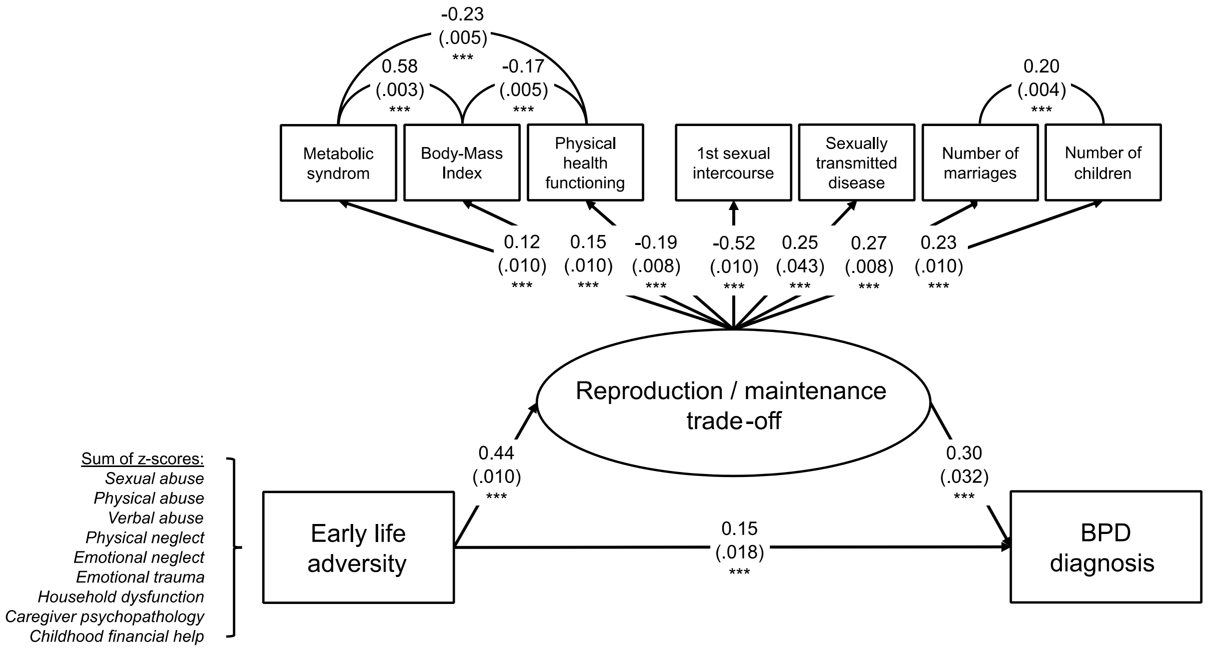

Latent Mediation Model for the effect of Early life adversity on the presence or absence of a BPD diagnosis. The ellipse represents the latent variable; rectangles represent its indicators. Early life adversity is modeled as a single composite variable, here represented by a rectangle. Paths between Early life adversity, the reproduction/maintenance trade-off latent factor and BPD diagnosis represent regressions. Paths between the indicators and the reproduction/maintenance trade-off latent factor represent factor loadings. Stars indicate significance: * for p<0.05, ** for p<0.01, and *** for p<0.001. CFI= 0.992; TLI=0.974; RMSEA= 0.018 (0.016 - 0.020); SRMR= 0.029

© 2023 Baptista A et al. *JAMA Psychiatry.*

eFigure 8. Inclusion of an additional adjustment for all lifetime Axis I and Axis II disorders

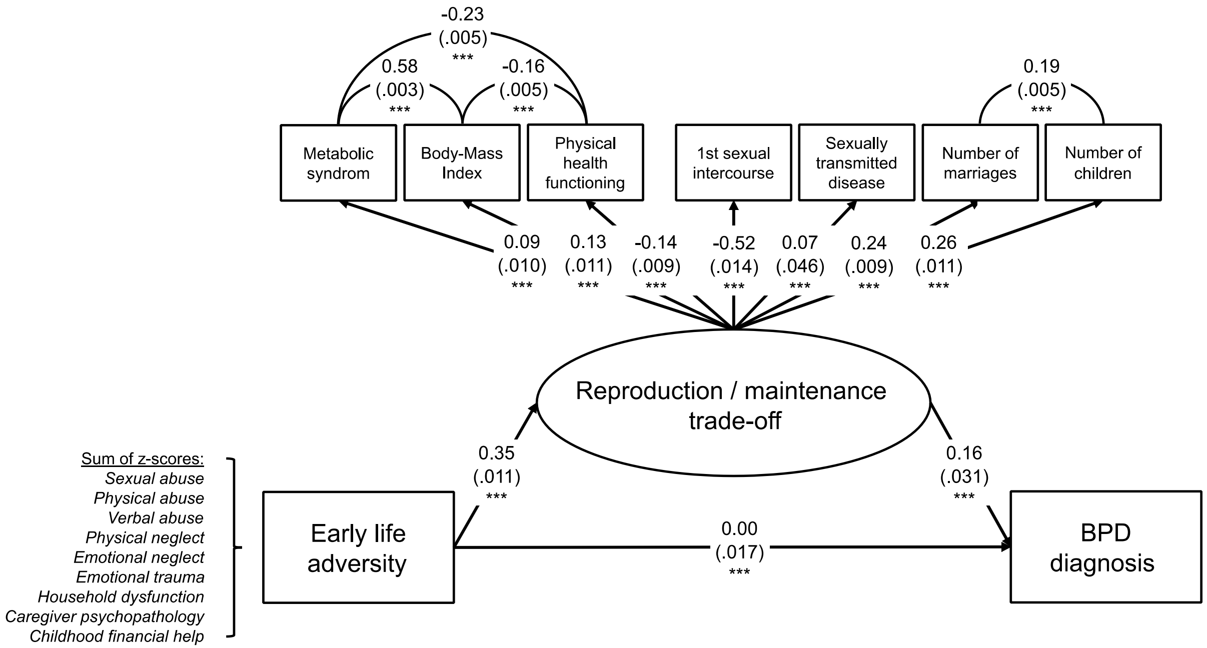

Latent Mediation Model for the effect of Early life adversity on the presence or absence of a BPD diagnosis. The ellipse represents the latent variable; rectangles represent its indicators. Early life adversity is modeled as a single composite variable, here represented by a rectangle. Paths between Early life adversity, the reproduction/maintenance trade-off latent factor and BPD diagnosis represent regressions. Paths between the indicators and the reproduction/maintenance trade-off latent factor represent factor loadings. Stars indicate significance: * for p<0.05, ** for p<0.01, and *** for p<0.001. CFI= 0.994; TLI= 0.929; RMSEA= 0.013 (0.011 0.015); SRMR= 0.011

© 2023 Baptista A et al. *JAMA Psychiatry.*

eFigure 9. Age limit below 14 years old for childhood adverse events

Early life adversity items included in this sensitivity analysis (all variables with age limit < 14): "ever serve as peacekeeper/relief worker in war zone/other terrorized area", "ever an unarmed civilian in war/revolution/military coup", "ever a refugee ", "ever in serious/life-threatening accident", "ever in serious fire, tornado, flood, earthquake or hurricane", " ever sexually assaulted, molested, raped or experienced unwanted sex ", "physically attacked/beaten/injured by parent/caretaker", "seriously neglected by parent/caretaker", "saw serious fights at home", "ever physically attacked/beaten/injured by spouse or romantic partner", "ever physically attacked/beaten/injured by anyone else ", "ever kidnapped or held hostage or as a pow ", "ever stalked by anyone ", " ever mugged, held up or threatened with a weapon ", "ever had someone close to you die in terrorist attack "," ever had someone close to you injured in terrorist attack?", "ever yourself injured in terrorist attack ", "ever yourself directly experience a terrorist attack", "ever yourself indirectly experience a terrorist attack, like watching on tv", "other than terrorist attack, ever see someone badly injured/killed or ever unexpectedly see a dead body", "other than terrorist attack, ever have someone close to you die unexpectedly ", "ever have someone close to you experience any other serious/life-threatening illness, accident or injury", "someone close to you ever have any other very stressful/traumatic experience ", "ever yourself have any other very stressful/traumatic experience".

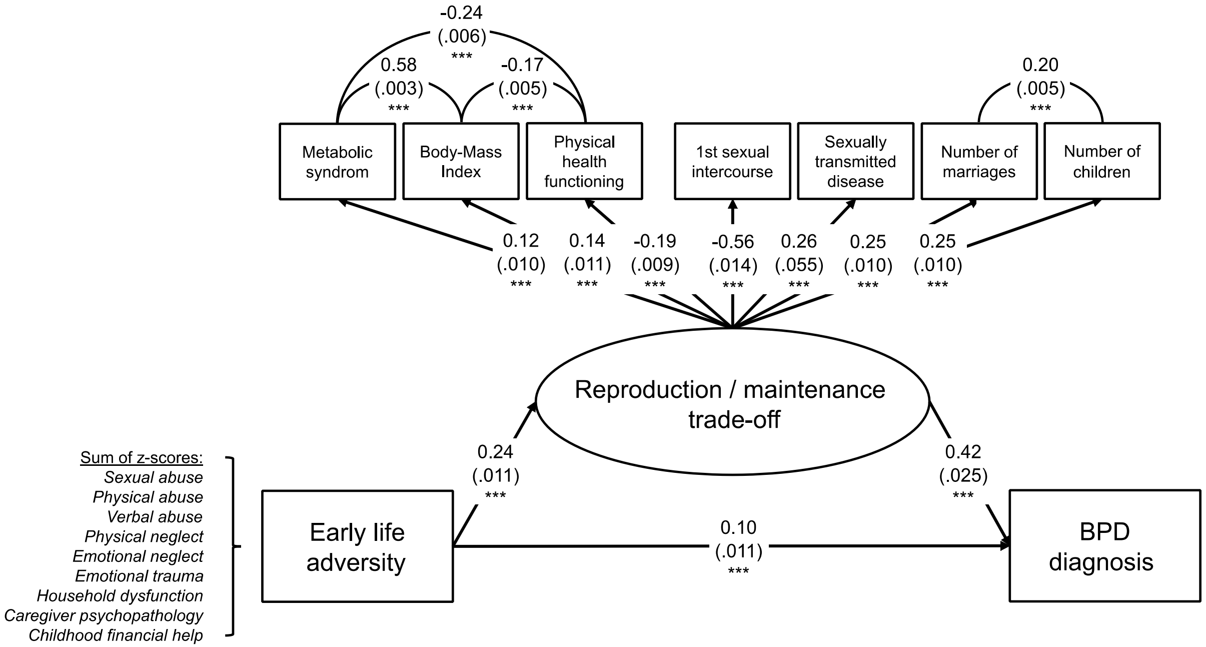

Latent Mediation Model for the effect of Early life adversity on the presence or absence of a BPD diagnosis. The ellipse represents the latent variable; rectangles represent its indicators. Early life adversity is modeled as a single composite variable, here represented by a rectangle. Paths between Early life adversity, the reproduction/maintenance trade-off latent factor and BPD diagnosis represent regressions. Paths between the indicators and the reproduction/maintenance trade-off latent factor represent factor loadings. Stars indicate significance: * for p<0.05, ** for p<0.01, and *** for p<0.001. CFI= 0.991; TLI= 0.976; RMSEA= 0.017 (0.015 0.019); SRMR = 0.030

© 2023 Baptista A et al. *JAMA Psychiatry.*

eMethods 6. 10-Fold cross validation

To test the capacity of our main model to generalize its predictions to out-of-sample data, we employed a 10-fold cross-validation procedure, following three main steps:

1. The full data set is randomly partitioned into 10 folds of nearly equal size.

2. Subsequently, 10 iterations of training and validation are performed such that within each iteration a different fold of the data is held-out for validation (i.e. the test data, here representing 10% of the whole sample) while the remaining k-1 folds are used for fitting (i.e. the training data, here representing 90% of the whole sample).

3. At each iteration, the model is fit on the training data and has its parameters fixed to these results.

Cross-validation performance was indexed by two measures: i) the global fit indices (RMSEA, CFI, TLI, SRMR statistics) which quantifies the adequacy of the fitted model to the test data (see eTable 8 right below), and ii) the Area Under the ROC Curve (AUC) which quantifies the capacity of the model to classify the presence or the absence of BPD in individual test data (see eFigure 10 below). Finally, the overall stability of these measures across the multiple partitioning of the dataset were used as indicators of the robustness of the results to sampling variability.

eTable 8. 10-Fold cross validation

Fold	X2 (train/test)	RMSEA (train/test)	CFI (train/test)	TLI (train/test)	SRMR (train/test)	ROC AUC (train/test)
1	277.657 / 219.009	0.021 (0.019 - 0.023)/ 0.025 (0.022 - 0.029)	0.989 / 0.942	0.969 / 0.953	0.033 / 0.076	0.8682 / 0.8961
2	248.342 / 157.693	0.019 (0.017 -0.022)/ 0.019 (0.015 - 0.024)	0.990 / 0.962	0.972 /0.969	0.026 / 0.090	0.8667 / 0.8554
3	236.893 / 149.773	0.019 (0.017 - 0.021)/ 0.018 (0.014 - 0.023)	0.991 / 0.974	0.974 / 0.979	0.032 / 0.071	0.8721 / 0.8786
4	248.959 / 297.343	0.019 (0.017 - 0.022)/ 0.032 (0.028 - 0.035)	0.990 / 0.914	0.972 / 0.931	0.031 / 0.068	0.8772 / 0.869
5	240.417 / 278.531	0.019 (0.017 -0.021) / 0.030 (0.027 -0.034)	0.990 / 0.930	0.973 / 0.943	0.032 / 0.101	0.8785 / 0.853
6	255.087 / 92.287	0.020 (0.018 - 0.022) / 0.009 (0.000 -0.014)	0.989 / 0.993	0.971 / 0.994	0.031 / 0.048	0.8753 / 0.8577
7	221.460 / 240.717	0.018 (0.016 -0.021) / 0.027 (0.024 -0.031)	0.991 /0.950	0.975 /0.959	0.030 / 0.069	0.8684 / 0.8816
8	225.139 / 123.172	0.018 (0.016 - 0.021) / 0.015 (0.010 - 0.019)	0.991 / 0.982	0.975 / 0.985	0.031 / 0.063	0.8753 / 0.876
9	243.070 / 153.699	0.019 (0.017 -0.021)/ 0.019 (0.015 - 0.023)	0.990 / 0.973	0.973 / 0.978	0.029 / 0.074	0.8758 / 0.8849
10	238.553 / 193.094	0.019 (0.017 - 0.021) / 0.023 (0.019 -0.027)	0.990 / 0.958	0.973 / 0.966	0.032 / 0.091	0.871 / 0.8536

© 2023 Baptista A et al. *JAMA Psychiatry.*

eFigure 10. 10-Fold cross validation: Area Under the Curve (AUC) of the Receiver Operating Characteristic (ROC) curves across train and test subsamples.

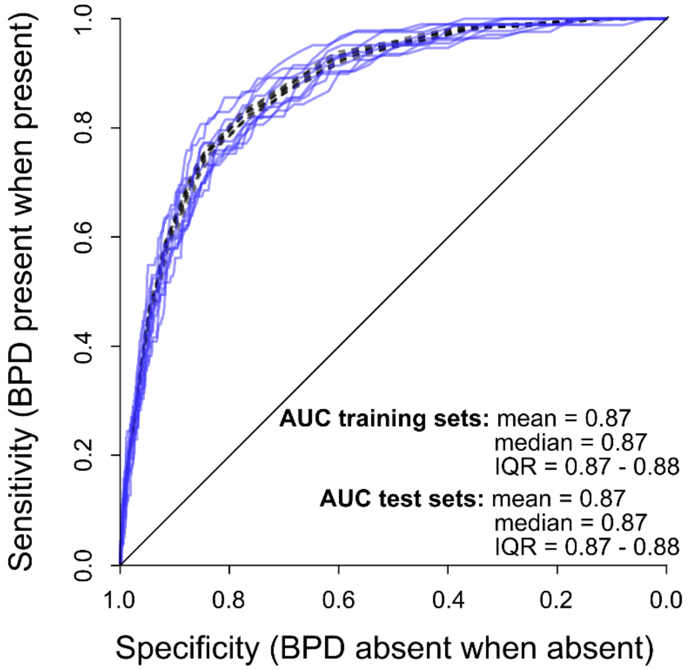

Sensitivity is the ability of the trained model to correctly identify the presence of a BPD diagnosis in the test subsamples. Specificity is the ability of the trained model to correctly identify the absence of a BPD diagnosis in the test subsamples. IQR = Interquartile Range.

© 2023 Baptista A et al. *JAMA Psychiatry.*

eReferences

1. Del Giudice M, Ellis BJ, Shirtcliff EA. The Adaptive Calibration Model of stress responsivity. *Neuroscience & Biobehavioral Reviews*. 2011;35(7):1562-1592. doi:10.1016/j.neubiorev.2010.11.007

2. Ruan WJ, Goldstein RB, Chou SP, et al. The Alcohol Use Disorder and Associated Disabilities Interview Schedule-IV (AUDADIS-IV): Reliability of new psychiatric diagnostic modules and risk factors in a general population sample. *Drug and Alcohol Dependence*. 2008;92(1-3):27-36. doi:10.1016/j.drugalcdep.2007.06.001

3. Straus MA. Measuring intrafamily conflict and violence: The conflict tactics (CT) scales. *Journal of Marriage and the Family*. Published online 1979:75-88.

4. Bernstein DP, Fink L, Handelsman L, et al. Initial reliability and validity of a new retrospective measure of child abuse and neglect. *The American journal of psychiatry*. Published online 1994.

5. Nettle D, Coall DA, Dickins TE. Birthweight and paternal involvement predict early reproduction in British women: evidence from the National Child Development Study. *American Journal of Human Biology: The Official Journal of the Human Biology Association*. 2010;22(2):172-179.

6. Simpson JA, Griskevicius V, Kuo SIC, Sung S, Collins WA. Evolution, stress, and sensitive periods: The influence of unpredictability in early versus late childhood on sex and risky behavior. *Developmental Psychology*. 2012;48(3):674-686. doi:10.1037/a0027293

7. Pepper GV, Nettle D. Out of control mortality matters: the effect of perceived uncontrollable mortality risk on a health-related decision. *PeerJ*. 2014;2:e459. doi:10.7717/peerj.459

8. Mell H, Safra L, Algan Y, Baumard N, Chevallier C. Childhood environmental harshness predicts coordinated health and reproductive strategies: A cross-sectional study of a nationally representative sample from France. *Evolution and Human Behavior*. 2018;39(1):1-8.

9. Ware Jr JE, Kosinski M, Keller SD. A 12-Item Short-Form Health Survey: construction of scales and preliminary tests of reliability and validity. *Medical care*. Published online 1996:220-233.

10. Muthén LK, Muthén B. *Mplus User's Guide: Statistical Analysis with Latent Variables, User's Guide*. Muthén & Muthén; 2017.

11. Fairchild AJ, McDaniel HL. Best (but oft-forgotten) practices: mediation analysis. *The American Journal of Clinical Nutrition*. 2017;105(6):1259-1271.

12. Muthén B, Asparouhov T. Causal Effects in Mediation Modeling: An Introduction With Applications to Latent Variables. *Structural Equation Modeling: A Multidisciplinary Journal*. 2015;22(1):12-23. doi:10.1080/10705511.2014.935843

13. MacKinnon DP, Lockwood CM, Hoffman JM, West SG, Sheets V. A comparison of methods to test mediation and other intervening variable effects. *Psychological Methods*. 2002;7(1):83-104. doi:10.1037/1082-989X.7.1.83

© 2023 Baptista A et al. *JAMA Psychiatry.*

14. Green DM, Swets JA. *Signal Detection Theory and Psychophysics*. Vol 1. Wiley New York; 1966.

15. Asparouhov T, Muthen B. Using Mplus individual residual plots for diagnostics and model evaluation in SEM. :16.

16. Asparouhov T, Muthén B. Simple second order chi-square correction. *Mplus technical appendix*. Published online 2010:1-8.

17. Vandenberg RJ, Lance CE. A review and synthesis of the measurement invariance literature: Suggestions, practices, and recommendations for organizational research. *Organizational research methods*. 2000;3(1):4-70.

18. Van de Schoot R, Lugtig P, Hox J. A checklist for testing measurement invariance. *European journal of developmental psychology*. 2012;9(4):486-492.

19. Hoertel N, López S, Wang S, González-Pinto A, Limosin F, Blanco C. Generalizability of pharmacological and psychotherapy clinical trial results for borderline personality disorder to community samples. *Personal Disord*. 2015;6(1):81-87. doi:10.1037/per0000091

© 2023 Baptista A et al. *JAMA Psychiatry.*
