# Supplementary material for: Associations Between Early Life Adversity, Reproduction-Oriented Life Strategy, and Borderline Personality Disorder
Source: JAMA Psychiatry. 2023 Apr 26;80(6):558–66. doi: 10.1001/jamapsychiatry.2023.0694 (PMC10134045; doi:10.1001/jamapsychiatry.2023.0694)
Supplement: Supplement 2. — Data sharing statement [file jamapsychiatry-e230694-s002.pdf]

Data Sharing Statement

Baptista. Associations Between Early Life Adversity, Reproduction-Oriented Life Strategy, and Borderline Personality Disorder. *JAMA Psychiatry*. Published April 26, 2023. doi:10.1001/jamapsychiatry.2023.0694

Data
Data available: No
